# Recruitment of Vps34 PI3K and enrichment of PI3P phosphoinositide in the viral replication compartment is crucial for replication of a positive-strand RNA virus

**DOI:** 10.1371/journal.ppat.1007530

**Published:** 2019-01-09

**Authors:** Zhike Feng, Kai Xu, Nikolay Kovalev, Peter D. Nagy

**Affiliations:** 1 Department of Plant Pathology, University of Kentucky, Lexington, Kentucky, United States of America; 2 Jiangsu Key Laboratory for Microbes and Functional Genomics, College of Life Sciences, Nanjing Normal University, Nanjing, China; The University of Chicago, UNITED STATES

## Abstract

Tombusviruses depend on subversions of multiple host factors and retarget cellular pathways to support viral replication. In this work, we demonstrate that tomato bushy stunt virus (TBSV) and the closely-related carnation Italian ringspot virus (CIRV) recruit the cellular Vps34 phosphatidylinositol 3-kinase (PI3K) into the large viral replication compartment. The kinase function of Vps34 is critical for TBSV replication, suggesting that PI(3)P phosphoinositide is utilized by TBSV for building of the replication compartment. We also observed increased expression of Vps34 and the higher abundance of PI(3)P in the presence of the tombusviral replication proteins, which likely leads to more efficient tombusvirus replication. Accordingly, overexpression of PI(3)P phosphatase in yeast or plants inhibited TBSV replication on the peroxisomal membranes and CIRV replication on the mitochondrial membranes. Moreover, the purified PI(3)P phosphatase reduced TBSV replicase assembly in a cell-free system. Detection of PI(3)P with antibody or a bioprobe revealed the enrichment of PI(3)P in the replication compartment. Vps34 is directly recruited into the replication compartment through interaction with p33 replication protein. Gene deletion analysis in surrogate yeast host unraveled that TBSV replication requires the vesicle transport function of Vps34. In the absence of Vps34, TBSV cannot efficiently recruit the Rab5-positive early endosomes, which provide PE-rich membranes for membrane biogenesis of the TBSV replication compartment. We found that Vps34 and PI(3)P needed for the stability of the p33 replication protein, which is degraded by the 26S proteasome when PI(3)P abundance was decreased by an inhibitor of Vps34. In summary, Vps34 and PI(3)P are needed for providing the optimal microenvironment for the replication of the peroxisomal TBSV and the mitochondrial CIRV.

## Author summary

Replication of RNA viruses infecting various eukaryotic organisms is the central step in the infection process that leads to generation of progeny viruses. The replication process requires the assembly of numerous viral replicase complexes within the large replication compartment, whose formation is not well understood. Using tombusviruses and the model host yeast, the authors discovered that a highly conserved cellular lipid kinase, Vps34 phosphatidylinositol 3-kinase (PI3K), is critical for the formation of the viral replication compartment. Expression of PI3K mutants and the PI(3)P phosphatase revealed that the PI(3)P phosphoinositide produced by Vps34 is crucial for tombusvirus replication. Tombusviruses co-opt Vps34 through interaction with the viral replication protein into the replication compartment. *In vitro* reconstitution of the tombusvirus replicase revealed the need for Vps34 and PI(3)P for the full-activity of the viral replicase. Chemical inhibition of Vps34 in yeast or plants showed that PI(3)P is important for the replication of several plant viruses within the Tombusviridae family and the insect-infecting Nodamuravirus. These results open up the possibility that the cellular Vps34 PI3K could be a target for new antiviral strategies.

## Introduction

Positive-strand RNA viruses replicate inside the infected plant or animal cells by utilizing subcellular membranes and co-opting multiple host proteins. These viruses generate membranous viral replication compartments, often harboring numerous vesicle-like membrane invaginations with narrow openings towards the cytosol [1–5]. The viral replication compartment help sequestering viral proteins, viral RNAs and co-opted host factors in confined areas, which facilitate efficient viral replicase complex (VRC) assembly and robust viral RNA replication. The replication compartment also protects the viral RNA from cellular defense mechanisms [5,6]. In spite of intensive research on the formation of viral replication compartments, it is still incompletely understood how the VRC assembly process is guided by viral and host factors.

The plant-infecting tombusviruses, such as Tomato bushy stunt virus (TBSV), induce complex rearrangements of cellular membranes, alter metabolic processes with the help of a number of co-opted host proteins [7–9]. The pro-viral host proteins include protein chaperones, translation elongation factors, DEAD-box helicases, glycolytic enzymes, the actin network, and cellular membrane remodeling proteins, such as the endosomal sorting complex required for transport (ESCRT) machinery [3,8,10–12]. TBSV also exploits sterols and phospholipids to induce a membranous replication compartment harboring numerous spherules, which are vesicle-like invaginations in the peroxisomal membranes [7,13–15].

Tombusviruses have a wide host range and they are among the best-characterized viruses [16–19]. They have one component (+)RNA genome of ~4.8 kb [20]. They belong to Flavivirus-like supergroup that includes important human, animal and plant pathogens. Tombusviruses code for five proteins including two essential replication proteins, p33 and p92^pol^, which is the RdRp protein and it is translated from the genomic RNA via readthrough of the translational stop codon in p33 ORF. The second replication protein, p33, is an RNA chaperone involved in recruitment of the viral (+)RNA for replication [20–22]. The TBSV replicon (rep)RNA, which is based on DI-72 RNA, contains four non-contiguous segments from the gRNA, can replicate efficiently in yeast and plant cells expressing p33 and p92^pol^ [20,23]. The replication of repRNA, which produces a double-stranded RNA replication intermediate, occurs in vesicle-like structures, called spherules in cells [7,8].

Intriguingly, tombusviruses take advantage of various cellular compartments for VRC assembly [24]. TBSV and the closely related cucumber necrosis virus (CNV) use peroxisomal membranes, whereas carnation Italian ringspot virus (CIRV) utilizes the outer membranes in mitochondria. The ER could support TBSV replication efficiently in the absence of peroxisomes in yeast [25,26]. Moreover, the formation of membrane contact sites (MCSs) between the ER and peroxisomes promote sterol-enrichment at replication sites [13,27]. Also, TBSV hijacks the Rab5-positive endosomes to build large replication compartments in yeast and plant cells [28].

### Role of Vps34 PI3K lipid kinase in the cell

Unlike mammals, yeast and plants have only one phosphatidylinositol (PI) 3-kinase (PI3K), namely Vps34, which produces phosphatidylinositol-3-phosphate, PI(3)P, a critical signaling and structural lipid molecule [29,30]. The multiple functions of Vps34 are to facilitate the formation of early endosomes involved in protein secretion and recycling; and autophagic structures involved in protein/lipid recycling during starvation [31–33]. Vps34 PI3K has attracted immense interest due to its role in human diseases, such as numerous forms of cancer, heart problems and neurodegeneration in humans [34,35]. The best-known role of Vps34 PI3K is in hepatitis C virus (HCV) replication [36–38]. Also, Rab5 GTPase and Vps34 are involved in HCV NS4B-induced autophagy [37]. Many DNA and RNA viruses are also influenced by the PI3K signaling pathway [39,40]. Since (+)RNA viruses remodel subcellular membranes to facilitate virus replication and avoid antiviral responses [3,9,41–44], it is possible that subversion of Vps34 PI3K and PI(3)P could be wide-spread among viruses.

### Role of PI(3)P phosphoinositide in the cell

Although PI(3)P is a minor lipid in the cell, it is a key player in endosomal vesicle trafficking by conferring identity to endosomes [29,30]. Also, PI(3)P plays a crucial role in regulating vesicle fusion and autophagosome formation. The accumulation of PI(3)P helps the recruitment of its numerous protein effectors. Many intracellular microbes and parasites exploit the cellular PI(3)P to establish infections. For example, the SopB effector of the *Salmonella* bacterium recruits Vps34 to the bacteria-containing vacuole, leading to enrichment of PI(3)P and maturation of the bacteria-containing vacuole [45]. *Mycobacterium tuberculosis*, *Phytophtora* and *Plasmodium* parasites also exploit PI(3)P to regulate endosomal functions [45,46]. In addition, elevated PI(3)P level induced artemisinin resistance in malaria parasites [47]. All these examples highlight the central roles of Vps34 and PI(3)P in microbe-host intracellular interactions.

In our previous paper, we showed the recruitment of the Rab5-positive endosomes to the large replication compartment formed in tombusvirus-infected cells [28]. Through specific interaction of the p33 replication protein with Rab5 small GTPase, tombusviruses enrich endosomal lipids, most importantly phosphatidylethanolamine (PE), but also PI(3)P, in peroxisomes or mitochondria for different tombusviruses. This raised the question if PI(3)P plays a role in tombusvirus replication. Accordingly, in this paper we show that TBSV hijacks Vps34p PI3K that leads to enrichment of PI(3)P in the large viral replication compartment in model yeast and plant hosts. Altogether, the direct interaction between p33 replication protein and Vps34p is needed for the biogenesis of the replication compartments.

## Results

### Deletion of *VPS34* PI3K inhibits viral replication in yeast

**Fig 1:**
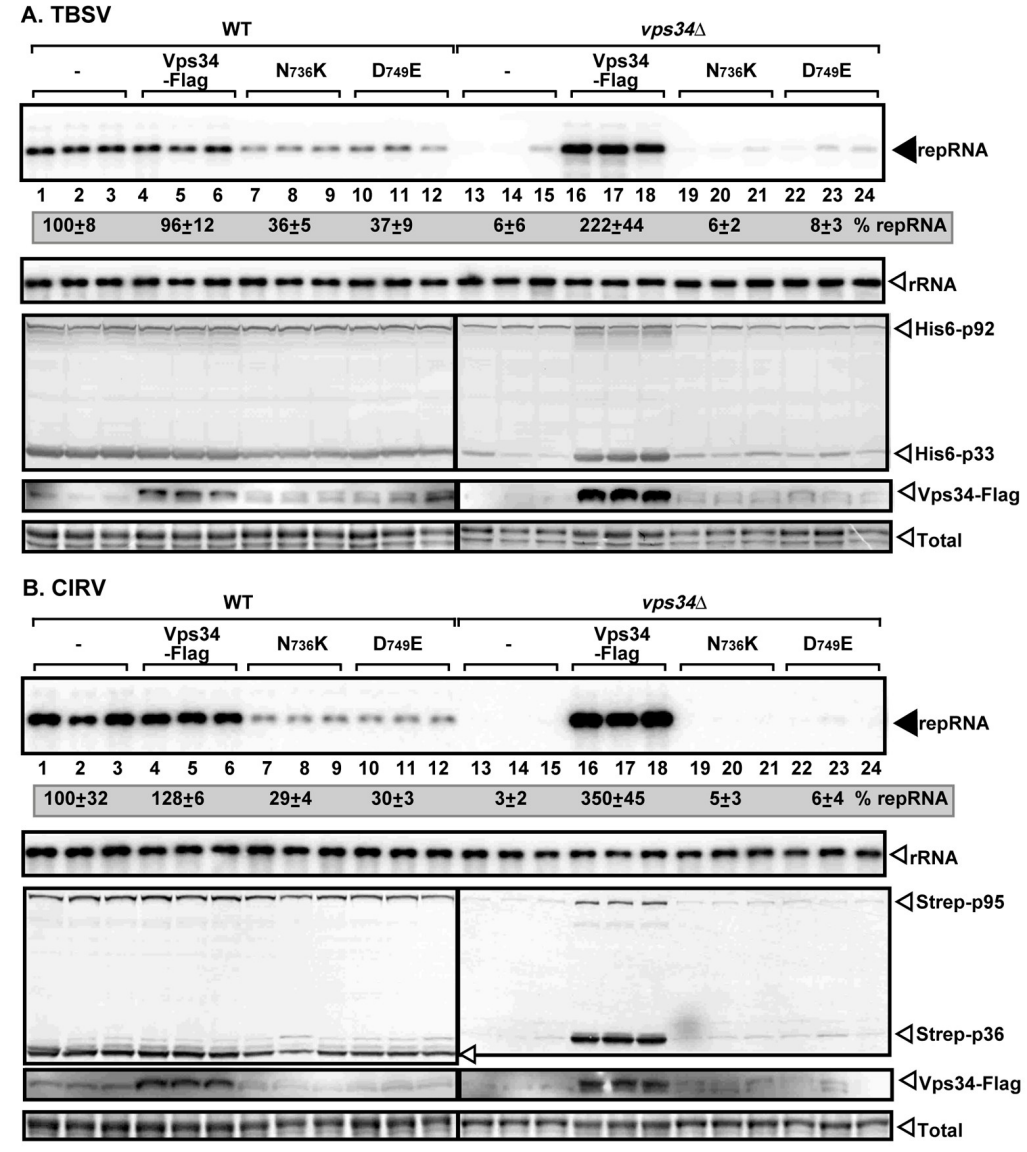
Vps34p PI3K is an essential host factor for tombusvirus replication in yeast. (A) Deletion of Vps34 inhibits TBSV replication in yeast. Northern blot analysis of TBSV repRNA using a 3’ end specific probe shows reduced accumulation of repRNA in vps34Δ yeast strain in comparison with the wt yeast strain. Viral proteins His_6_-p33 and His_6_-p92 were expressed from plasmids from the galactose-inducible *GAL1* promoter, while DI-72(+) repRNA was expressed from the *GAL10* promoter. Vps34-Flag and its two defective mutants were separately expressed from a plasmid as shown. Northern blot with 18S ribosomal RNA specific probe was used as a loading control. Bottom images: Western blot analysis of the level of His_6_-p33 and His_6_-p92 with anti-His antibody and Vps34p with anti-Flag antibody. (B) Reduced CIRV replication in vps34Δ yeast strain. CIRV proteins Strep-p36 and Strep-p95 were expressed from plasmids from the *GAL1* promoter. See further details in panel A.

The recruitment of the components of the early endosome to the tombusvirus replication compartment leads to enrichment of PI(3)P within the replication sites [28], suggesting that PI(3)P phosphoinositide might be involved in the viral replication process. To study the putative role of PI(3)P in tombusvirus replication, first we analyzed if Vps34 PI3K, which is the only PI(3)P kinase in yeast [31,48,49], affects TBSV replication in yeast. We launched TBSV replication in a yeast strain lacking *VPS34* gene (vps34Δ) by expressing the p33 and p92^pol^ replication proteins and the repRNA from plasmids, followed by measuring TBSV repRNA level by Northern blotting. These experiments demonstrated that TBSV replicated only at a ~6% level in the absence of Vps34 protein when compared to the replication level in the WT yeast (Fig 1A, lanes 13–15 versus 1–3). Interestingly, the p33 replication protein accumulated poorly in vps34Δ yeast (Fig 1A). Complementation of tombusvirus replication by expression of wt Vps34p from a plasmid in vps34Δ yeast supported 2-fold higher level of viral repRNA accumulation than in WT yeast (Fig 1A, lanes 16–18), demonstrating pro-viral function for TBSV replication. Interestingly, expression of two inactive forms of Vps34 (N_736_K and D_749_E) [49,50], which are defective in producing PI(3)P, in vps34Δ yeast could not complement the pro-viral function (Fig 1A, lanes 19–24), suggesting that PI(3)P is required for TBSV replication. Moreover, the expression of the two inactive mutant forms of Vps34 inhibited TBSV repRNA accumulation in WT yeast (Fig 1A, lanes 7–12 versus 1–3), likely due to the competition between the WT Vps34p expressed from the chromosome and the Vps34p mutants expressed from plasmids to participate in protein complexes (see below).

To test if the closely-related CIRV, which replicates on the outer mitochondrial membranes, also requires Vps34p, we measured repRNA replication when the CIRV replication proteins were expressed in vps34Δ yeast. The accumulation of repRNA in yeast decreased by ~30-fold in the absence of Vps34p (Fig 1B, lanes 13–15), confirming that CIRV also requires the pro-viral functions of Vps34p. Expression of the plasmid-borne Vps34p in vps34Δ yeast increased repRNA accumulation by ~3.5-fold in comparison with wt yeast (Fig 1B, lanes 16–18), whereas N_736_K and D_749_E mutants of Vps34p behaved as dominant negatives in viral replication when expressed in WT yeast (Fig 1B, lanes 7–12). Both p36 and p95^pol^ replication proteins showed reduced accumulation in vps34Δ yeast (Fig 1B). Overall, we conclude that Vps34 provides critical pro-viral functions for both the peroxisomal TBSV and the mitochondrial CIRV replication in yeast cells.

**Fig 2:**
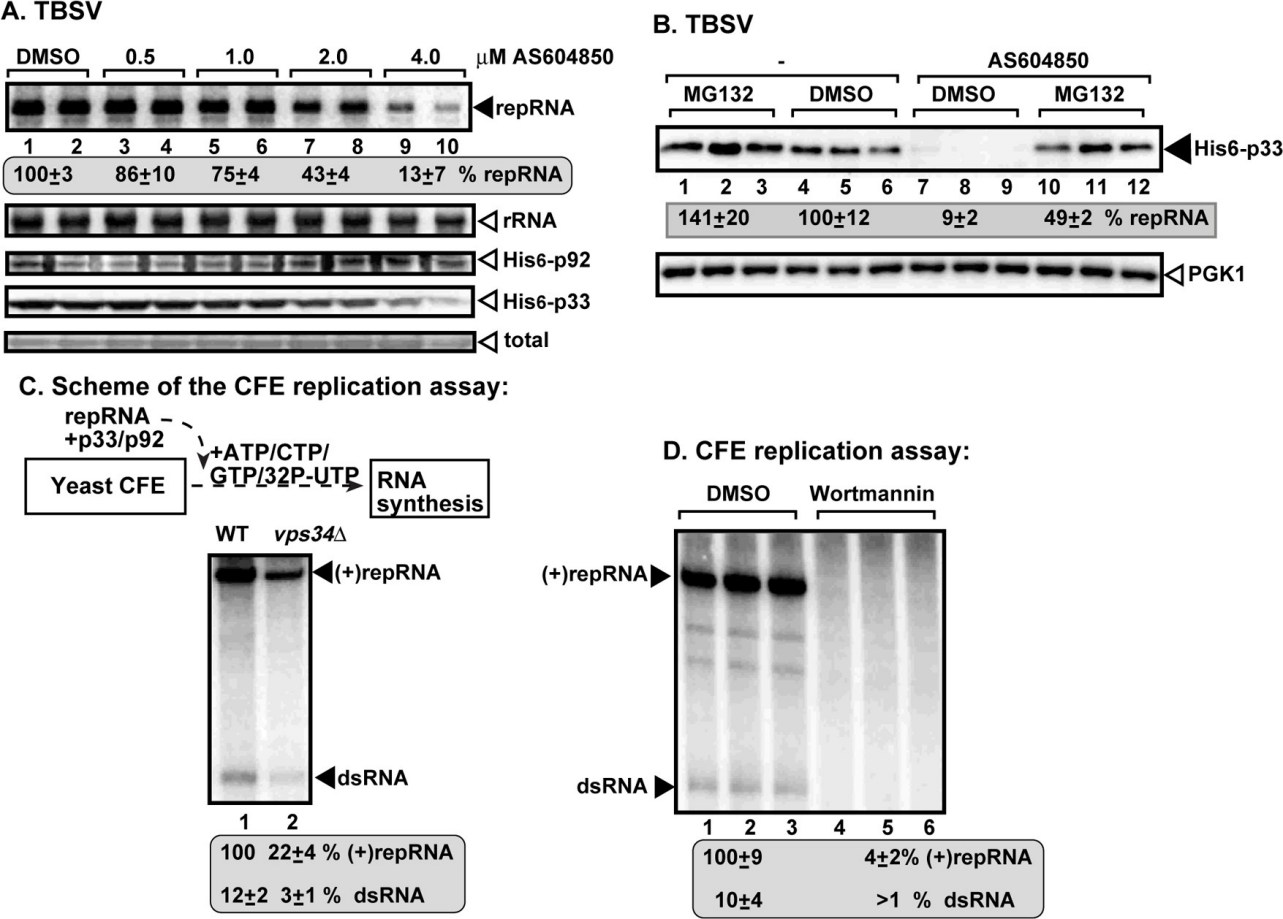
Chemical inhibitor of Vps34 inhibits TBSV replication and decreases the stability of the viral replication protein. (A) Inhibition of TBSV replication by the PI3K inhibitor AS604850 in yeast. See further details in Fig 1A. (B) Western blot analysis of TBSV p33 replication protein level in the presence or absence of AS604850 inhibitor. Yeast spheroplasts expressing p33 were treated with MG132 proteasome inhibitor or DMSO as shown. As a loading control, the cellular PGK1 was detected with anti-PGK1 antibody (bottom panel). The samples were taken after 2 h of treatments of spheroplasts. (C) Reduced TBSV RNA production by the tombusvirus replicase assembled *in vitro* in cell-free extract (CFE) prepared from vps34Δ yeast in comparison with a similar CFE preparation obtained from wt yeast. Purified recombinant p33 and p92^pol^ replication proteins of TBSV and *in vitro* transcribed TBSV DI-72 (+)repRNA were added to the CFEs. Nondenaturing PAGE analysis shows the ^32^P-labeled TBSV repRNA products, including the (+)repRNA progeny and the dsRNA replication intermediate, made by the reconstituted TBSV replicase. (D) Reduced TBSV RNA production by the tombusvirus replicase assembled *in vitro* in CFEs prepared from Wortmannin or DMSO-treated WT yeast. See further details in panel C above. Each experiment was repeated.

To obtain further evidence on the pro-viral role of Vps34p, we used a specific inhibitor (AS604850) [51] to block Vps34 activity in yeast cells. We found ~8-fold reduction in TBSV repRNA accumulation when the highest concentration of the inhibitor was used (Fig 2A). Similar to the deletion of *VPS34*, chemical inhibition of Vps34p activity also resulted in reduced accumulation of p33 replication protein in WT yeast (Fig 2A). Similar studies using AS604850 inhibitor of Vps34p showed that replication of CIRV, the closely related cucumber necrosis virus (CNV) and the unrelated Nodamura virus (NoV, an insect-infecting alfanodavirus) is also dependent on Vps34p functions in yeast cells (S1 Fig). The NoV RNA1 replication was more sensitive to AS604850 inhibitor than the transcription of subgenomic RNA3, which was decreased only when the largest amount of inhibitor was applied (S1C Fig)

To test the fate of p33 replication protein when Vps34 activity is blocked, we used MG132 inhibitor to block the function of the 26S proteasome in yeast [52]. We found a 40% increased level of p33 replication protein in MG132-treated versus DMSO-treated wt yeast spheroplasts (Fig 2B). Moreover, MG132-treatment also reversed the negative effect of AS604850 inhibitor on the p33 level by resulting in ~5-fold higher level p33 in yeast spheroplasts (Fig 2B), suggesting that p33 becomes degraded by the proteasome when Vps34 function is inhibited and PI(3)P is not produced in the viral replication compartment.

Testing the activity of the *in vitro* assembled tombusvirus replicase based on purified recombinant replication proteins in yeast cell-free extracts (CFEs) revealed ~5-fold reduced activities for CFEs obtained from vps34Δ yeast in comparison with wt yeast CFEs (Fig 2C). The accumulation of both the dsRNA replication intermediate and the newly-made (+)RNA progeny decreased in CFEs prepared from vps34Δ yeast, indicating that Vps34 activity is likely required during the replicase complex assembly step *in vitro*. This was further supported by another CFE-based replicase assembly experiment when WT yeast was grown in the presence of Wortmannin, an inhibitor of Vps34p (Fig 2D). These results also suggest that Vps34p PI3K activity is required not only for maintaining p33 replication protein level in yeast, but during the actual replication process as well. This is because we provided the same amounts of purified recombinant replication proteins for the above CFE-based assays (Fig 2C and 2D) [53]. Thus, these *in vitro* results with Vps34p are different from the Rab5-based studies [28], suggesting that the pro-viral role of Vps34p is more complex than that of co-opted Rab5 and the endosomes in tombusvirus replication.

### Vps34 PI3K has pro-viral functions in plants

**Fig 3:**
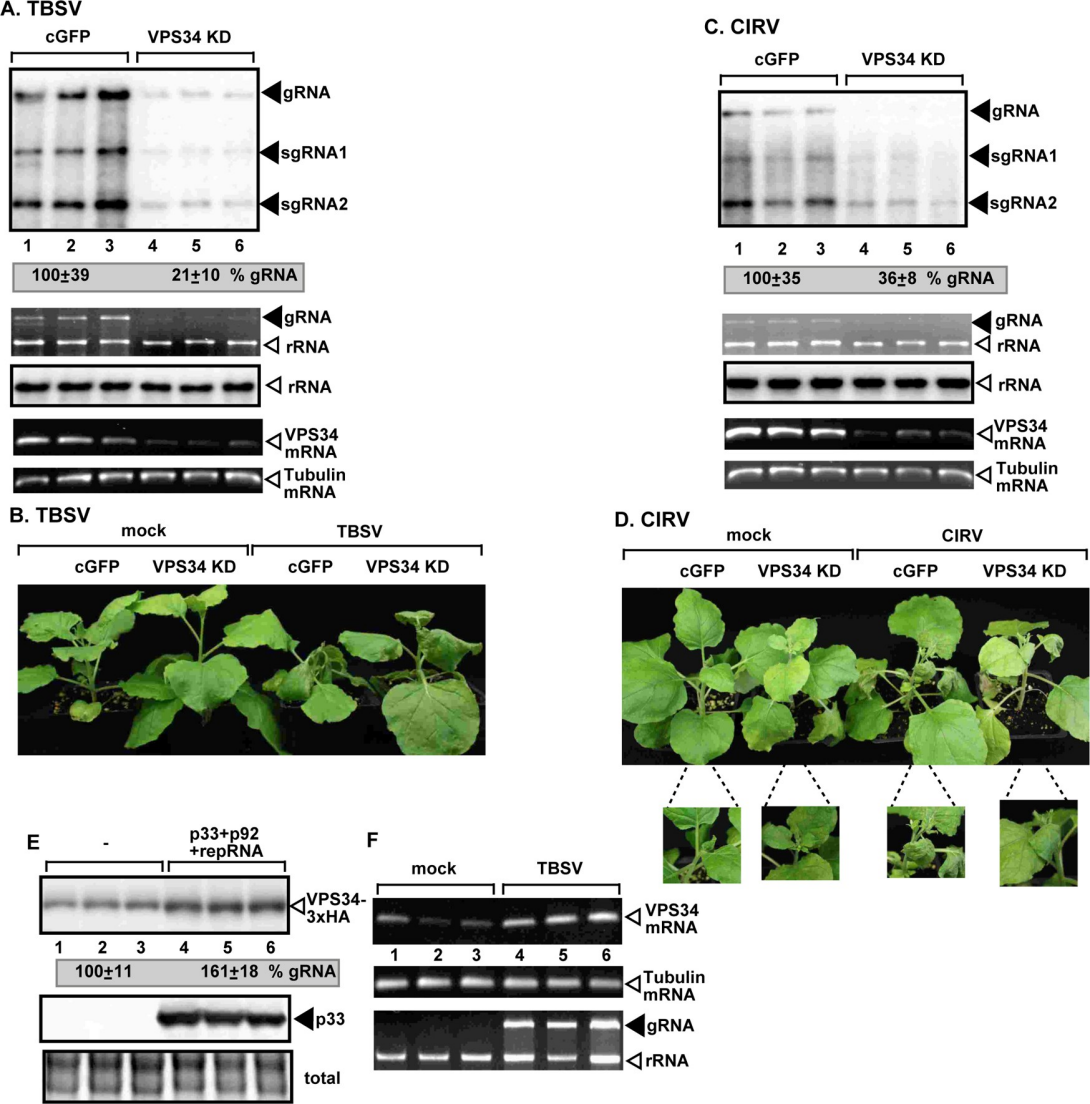
Knockdown of VPS34 gene expression inhibits tombusvirus replication in *N*. *benthamiana* plants. (A) Top panel: Accumulation of the TBSV genomic (g)RNA in VPS34- silenced *N*. *benthamiana* plants 2 days post-inoculation (dpi) was measured by Northern blot analysis. Inoculation of TBSV gRNA was done 12 days after silencing of *VPS34* expression. VIGS was performed via agroinfiltration of tobacco rattle virus (TRV) vector carrying NbVps34 or 3’-terminal GFP (as a control) sequences. Second panel: Ribosomal RNA is shown as a loading control in an ethidium-bromide stained agarose gel. Note that the TBSV genomic RNA is also visible in the gel. Third panel: Northern blot with *N*. *benthamiana* 18S ribosomal RNA specific probe was used as a loading control. Fourth panel: RT-PCR analysis of NbVps34 mRNA level in the silenced and control plants. Fifth panel: RT-PCR analysis of *TUBULIN* mRNA level in the silenced and control plants. Each experiment was repeated. (B) Delayed development of TBSV-induced symptoms is observed in *VPS34*-silenced *N*. *benthamiana* plants as compared with the control plants. Note the lack of phenotype in *VPS34*-silenced *N*. *benthamiana* plants. The picture was taken 5 dpi. (C) Top panel: Accumulation of the CIRV genomic (g)RNA in VPS34-silenced *N*. *benthamiana* plants 2.5 dpi was measured by Northern blot analysis. See further details in panel A. (D) Delayed development of CIRV-induced symptoms is observed in *VPS34*-silenced *N*. *benthamiana* plants as compared with the control plants. The picture was taken 5 dpi. (E) Western blot analysis of Vps34 level in yeast replicating TBSV repRNA or lacking viral components. The HA-tagged Vps34p was expressed from its natural promoter and original chromosomal location. The 6xHis-tagged p33 was detected by anti-His antibody. (F) Top panel: Induction of VPS34 mRNA expression in *N*. *benthamiana* plants infected with TBSV was detected by semi-quantitative RT-PCR. Middle panel: RT-PCR of tubulin mRNA was used as a control. Bottom panel: Ribosomal RNA is shown as a loading control in an ethidium-bromide-stained agarose gel.

To explore if tombusviruses depend on Vps34p functions in plants, first we knocked down Vps34 level based on gene-silencing in *N*. *benthamiana* plants. Similar to yeast, plants also have only a single PI3K kinase, namely Vps34 [54]. Knockdown of Vps34 in *N*. *benthamiana* led to ~5-fold reduction of TBSV genomic (g)RNA and ~3-fold reduction in CIRV RNA accumulation, respectively (Fig 3A–3C, lanes 4–6). These findings confirmed the pro-viral function of Vps34 in tombusvirus replication. Knockdown of Vps34 delayed the symptom formation and necrosis in young leaves infected with TBSV or CIRV (Fig 3B–3D). Second, we measured the amount of Vps34p, which is increased by 60% from its native promoter and original chromosomal location upon expression of p33 and p92 replication proteins in WT yeast (Fig 3E). In addition, we analyzed Vps34 mRNA levels in TBSV-infected versus mock-treated *Nicotiana benthamiana* leaves. RT-PCR results showed up-regulation of Vps34 mRNA level in TBSV-infected leaves (Fig 3F), suggesting that TBSV replication induces increased Vps34 expression in plants and yeast.

### Tombusviruses recruit Vps34 PI3K into the viral replication compartment in yeast and plant cells

**Fig 4:**
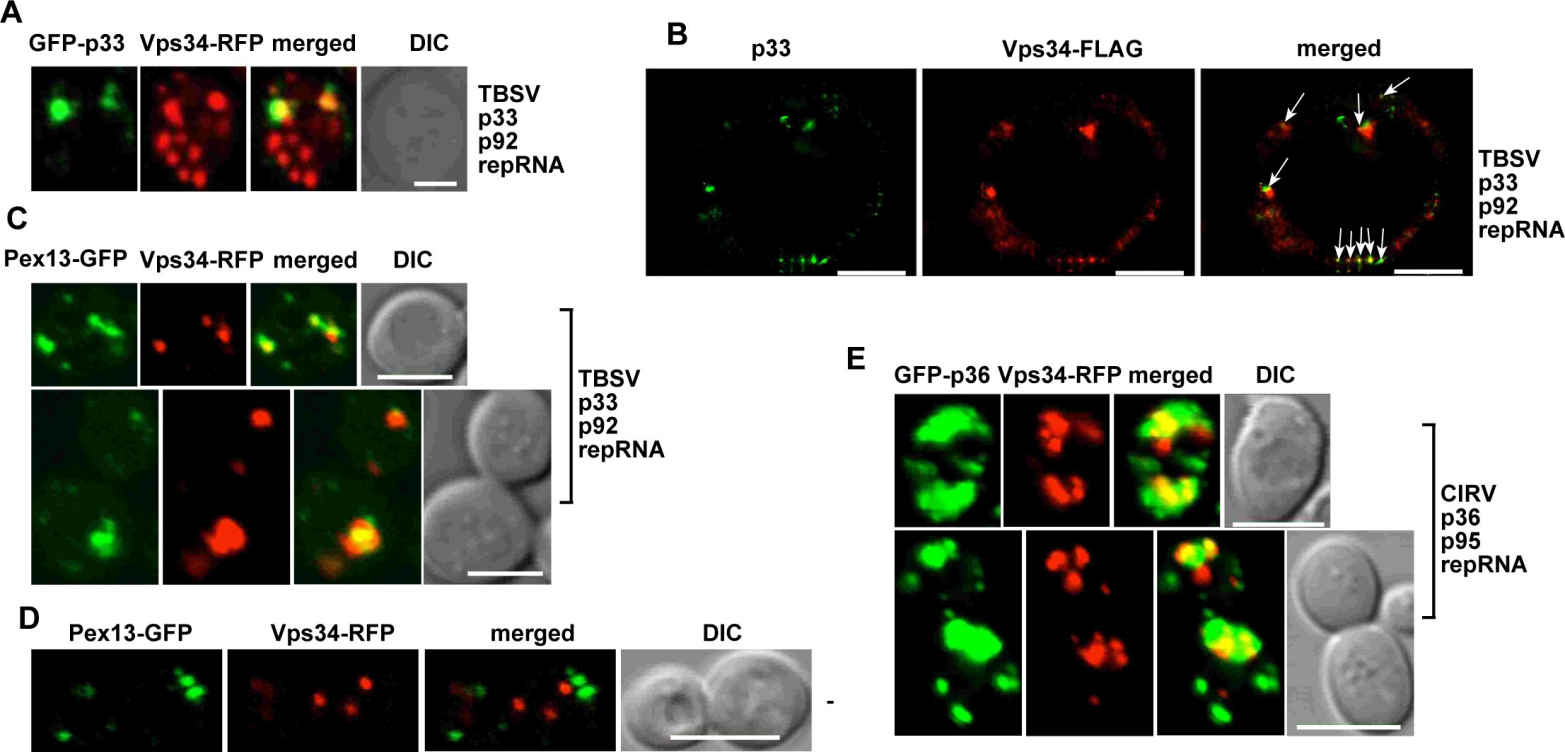
Co-localization of tombusvirus replication protein with Vps34p in yeast. (A) Confocal laser microscopy images show the partial co-localization of TBSV GFP-tagged p33 replication protein with the RFP-tagged Vps34p protein in wt yeast cells. DIC (differential interference contrast) images are shown on the right. Scale bar represents 2 μm. (B) Super-resolution microscopy images show the partial co-localization of TBSV p33 replication protein with Vps34-FLAG in yeast. Note that p33 was detected with anti-p33 antibody, whereas Vps34 was detected via anti-Flag antibody. Arrows point at the areas where co-localization is observed. Scale bars represent 1 μm. (C) Vps34p is recruited to the large replication compartments consisting of aggregated peroxisomes. The peroxisomes are marked with Pex13-GFP. Scale bars represent 5 μm. (D) Lack of co-localization of Vps34 and Pex13 in the absence of viral replication. See further details in panel C. Scale bars represent 5 μm. (E) Confocal laser microscopy images show the partial co-localization of CIRV GFP-p36 replication protein with Vps34-RFP in yeast. Scale bars represent 5 μm. See further details in panel A.

To learn if Vps34p lipid kinase is co-opted by TBSV for supporting its replication, first, we co-expressed RFP-tagged Vps34p with GFP-tagged TBSV p33 replication protein in wt yeast cells, followed by confocal imaging. These experiments revealed partial co-localization of TBSV p33 replication protein and Vps34 (71±26% co-localization from the p33 point of view, Fig 4A). Partial co-localization of FLAG-tagged Vps34 and p33 (using anti-p33 antibody) in yeast was also observed using super-resolution microscopy (Fig 4B). Co-expression of Vps34-RFP with Pex13-GFP peroxisomal marker protein in the presence of replicating TBSV replicon (rep)RNA in yeast also showed partial co-localization pattern (Fig 4C), whereas Vps34-RFP and Pex13-GFP did not co-localize in the absence of viral components (Fig 4D). The co-localization of Pex13-GFP and p33 replication protein is complete within the viral replication compartment consisting of aggregated peroxisomes [55]. In addition, similar partial co-localization pattern was observed when the GFP-p36 replication protein of the closely related CIRV, which localizes to the outer mitochondrial membranes [56], was co-expressed with Vps34-RFP in yeast cells (Fig 4E). Based on these experiments, we conclude that Vps34p lipid kinase is partially retargeted by tombusvirus replication proteins to the tombusvirus replication compartment in yeast.

**Fig 5:**
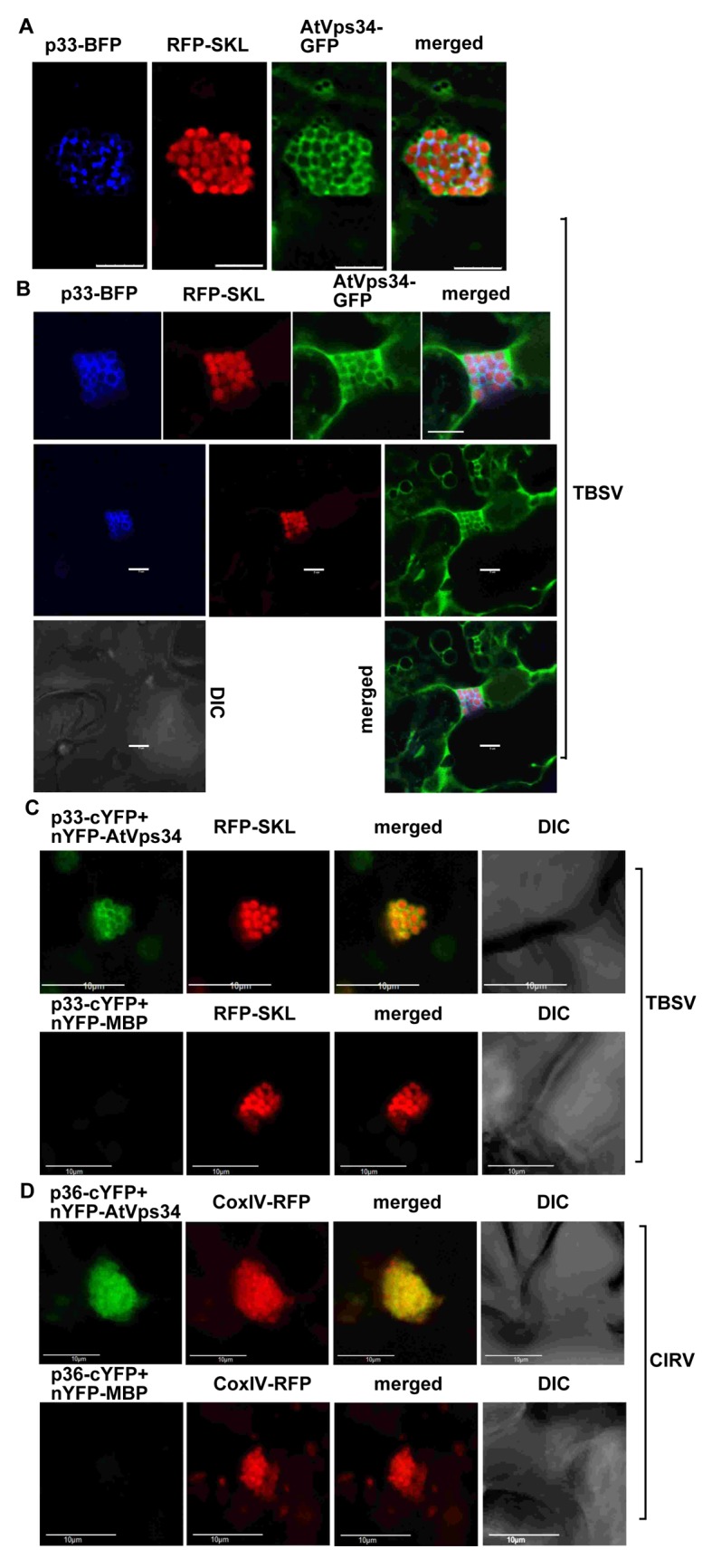
Recruitment of Vps34 by the TBSV p33 replication protein into the viral replication compartment in *N*. *benthamiana*. (A-B) Confocal microscopy images show co-localization of TBSV p33-BFP replication protein and the AtVps34-GFP, the ortholog of the yeast Vps34 protein, *in planta*. The large replication compartment was visualized via expression of RFP-SKL peroxisomal matrix marker protein. Expression of the above proteins from the 35S promoter was done after co-agroinfiltration into *N*. *benthamiana* leaves. Scale bars represent 5 μm. Note that the top panel in panel B is enlargement of the replication compartment portion of the bottom images. (C) Top images: interaction between TBSV p33-cYFP replication protein and the nYFP-AtVps34 protein was detected by BiFC. Co-localization of RFP-SKL with the BiFC signal (see merged image) demonstrates that the interaction between p33 replication protein and Vps34 occurs in the large viral replication compartments *in planta*. Control BiFC experiments included p33-cYFP protein in combination with nYFP-MBP protein expressed in *N*. *benthamiana* infected with TBSV (bottom images). Scale bars represent 10 μm. (D) Top images: interaction between CIRV p36-cYFP replication protein and the nYFP-AtVps34 protein was detected by BiFC. The CIRV replication compartment was visualized with CoxIV-RFP mitochondria marker. Scale bars represent 10 μm. See further details in panel C.

To confirm that comparable outcomes take place in the native plant cells, we co-expressed TBSV p33-BFP with the *Arabidopsis* AtVps34 and RFP-SKL (peroxisomal luminar marker protein) in *N*. *benthamiana* leaves infected with TBSV. Confocal microscopy imaging revealed the partial co-localization of AtVps34 with p33-BFP and RFP-SKL (Fig 5A and 5B), suggesting that Vps34 is partially recruited into the large TBSV replication compartment in plant cells. On the contrary, the peroxisomes did not show significant co-localization with AtVps34 in the absence of TBSV infection (S2 Fig).

### Tombusviruses replication proteins interact with the host Vps34 lipid kinase

To confirm that the plant Vps34 is recruited into the viral replication compartment through the interaction with the TBSV p33 or CIRV p36 replication proteins, we have performed bimolecular fluorescence complementation (BiFC) experiments in *N*. *benthamiana* leaves. The BiFC assay using confocal microscopic imaging revealed the interaction between Vps34 and p33/p36 replication proteins within the replication compartment, consiting of either aggregated peroxisomes or mitochondria (detected with the help of peroxisomal RFP-SKL and the mitochondrial CoxIV-RFP) (Fig 5C and 5D).

**Fig 6:**
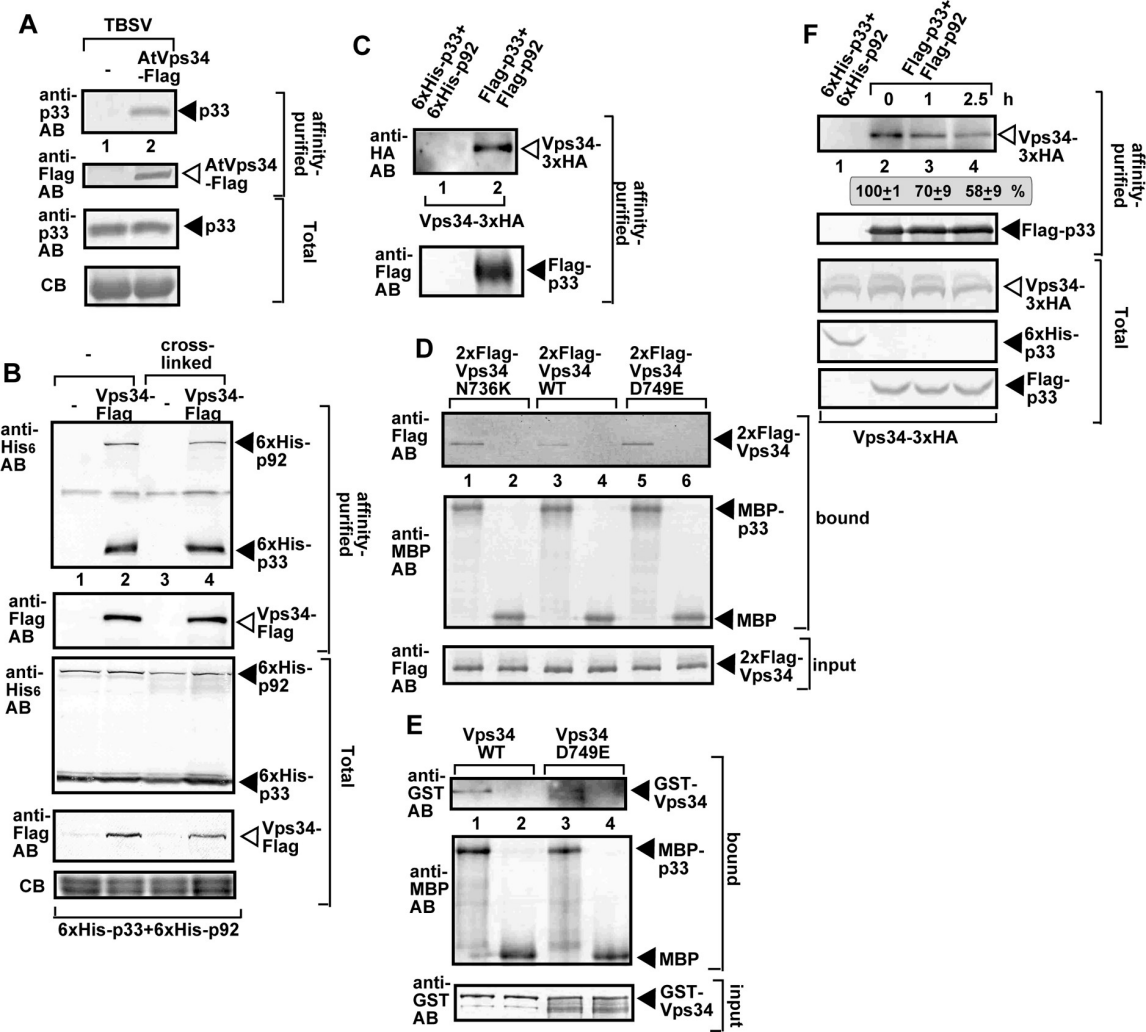
Interaction between p33 replication protein and Vps34p. (A) Co-purification of p33 replication protein with the AtVps34-Flag from subcellular membranes of *N*. *benthamiana* infected with TBSV. Top two panels: Western blot analysis of co-purified p33 (lane 2) with Flag-affinity purified AtVps34-Flag. P33 was detected with anti-p33 antibody, while AtVps34-Flag was detected with anti-FLAG antibody as shown. Bottom two panels: Western blot of total p33 and AtVps34-Flag in the total plant extracts. (B) Co-purification of p33 and p92^pol^ replication proteins with the yeast Vps34-Flag from subcellular membranes. Top two panels: Western blot analysis of co-purified His_6_-tagged p33 and His_6_-p92^pol^ (lanes 2 and 4) with Flag-affinity purified Vps34-Flag. His_6_-p33 and His_6_-p92^pol^ were detected with anti-His antibody, while Vps34-Flag was detected with anti-FLAG antibody. The negative control was from yeast expressing Flag peptide and His_6_-p33 and His_6_-p92^pol^ purified in a FLAG-affinity column (lanes 1 and 3). Samples were cross-linked or untreated as shown. Middle two panels: Western blot of total His_6_-p33 and His_6_-p92^pol^ and Vps34-Flag in the total yeast extracts. Bottom panel: SDS-PAGE analysis of total yeast extract using Coomassie blue staining. (C) Western blot analysis of co-purified 3xHA-tagged Vps34 (lanes 2) with Flag-affinity purified Flag-p33 and Flag-p92^pol^. Vps34-3xHA was expressed in yeast from its native promoter and original chromosomal location. Vps34-3xHA was detected with anti-HA antibody. The samples were not cross-linked. See further details in panel B above. (D) Pull-down assay including the 2xFlag-tagged yeast Vps34 and its inactive mutants and the MBP-tagged TBSV p33 replication protein. Top panel: Western blot analysis of the captured Vps34p expressed in yeast with the MBP-affinity purified p33 (purified from *E*. *coli*) was performed with anti-Flag antibody. The negative control was MBP (lanes 2, 4 and 6). Middle panel: Western blot analysis of the captured MBP-p33 and MBP was performed with anti-MBP antibody. Bottom panel: Western blot analysis of the total 2xFlag-Vps34p in total extracts. (E) Pull-down assay including the GST-tagged yeast Vps34 and its inactive mutant and the MBP-tagged TBSV p33 replication protein. Top panel: Western blot analysis of the captured GST-Vps34p expressed in *E*. *coli* with the MBP-affinity purified p33 (from *E*. *coli*) was performed with anti-GST antibody. See further details in panel D above. Note that the recombinant GST-Vps34 was purified here, whereas 2xFLAG-Vps34 was present in the soluble fraction of yeast CFE in panel D. (F) Co-purification of Vps34p with the viral replicase shows temporal association. The presence of Vps34p in the membrane-bound viral replicase was tested after blocking cellular translation by cycloheximide. The yeast samples were collected at the shown time points after the addition of cycloheximide to the yeast culture. Note that samples were from yeasts replicating TBSV repRNA. Top panel: Western blot analysis of co-purified 3xHA-tagged Vps34p with Flag-affinity purified Flag-p33 and Flag-p92^pol^ from membrane fraction of yeast. Vps34p was detected with anti-HA antibody. The negative control was His_6_-tagged p33 and His_6_-p92^pol^ purified from yeast extracts using a Flag-affinity column. Middle panel: Western blot of purified Flag-p33 detected with anti-Flag antibody. Bottom panels: Western blots of 3xHA-tagged Vps34p, His_6_-p33 (lane 1) and Flag-p33 proteins in the total yeast extracts using anti-HA, anti-His and anti-Flag antibodies. Each experiment was repeated three times.

To further confirm the interaction between the plant Vps34 and the TBSV replication protein, we agroinfiltrated *N*. *benthamiana* leaves to express the Flag-tagged AtVps34, followed by inoculation of the agroinfiltrated leaves with sap containing TBSV. Flag-affinity purification of AtVps34 from the detergent-solubilized membrane fraction resulted in detection of the co-purified p33 replication protein via anti-p33 antibody using Western blotting (Fig 6A, lane 2).

Since the above experiments indicated that Vps34 is recruited to the viral replication compartment in yeast, we also tested interaction between the replication proteins and the yeast Vps34p using co-purification experiments with Flag-tagged Vps34 and His_6_-taged TBSV replication proteins from yeast. Western-blot analysis of the Flag-affinity purified Vps34 samples showed the efficient co-purification of both p33 and p92 replication proteins from the membrane fraction of yeast (Fig 6B). To exclude that Vps34p could only be part of the tombusvirus replicase complex when over-expressed, we performed the reciprocal approach by Flag-affinity purification of Flag-p33 from the membrane fraction of yeast expressing Vps34-3xHA from its natural promoter and the original chromosomal location in wt yeast. These experiments confirmed that Vps34-3xHA could be co-purified with the TBSV replicase (Fig 6C, lane 2). Thus, the co-purification experiments supported interaction between the tombusvirus replication proteins and Vps34p PI3K.

We then performed pull-down experiments with purified recombinant p33 replication protein, which also supported direct interaction with Vps34p expressed in yeast (Fig 6D). The kinase inactive mutants of Vps34p bound to p33 in the pull-down assay, suggesting that the kinase activity is not needed for this interaction (Fig 6D). To test if the interaction between p33 replication protein and Vps34p depends on Rab5, which is also present in the early endosomes and interacts with p33 [28], we performed pull down experiments with purified recombinant MBP-p33 and GST-Vps34p expressed in *E*. *coli*, which does not have Rab5. These experiments also showed direct interaction between p33 and Vps34p and the kinase inactive mutant of Vps34p (Fig 6E). Vps21p, a Rab5 protein in yeast was co-purified with FLAG-p33 from the membrane fraction of yeast lacking *VPS34* gene, suggesting that p33 replication protein could interact separately with Vps21p and Vps34p (S3 Fig). Altogether, all these data support direct interaction between the tombusvirus replication proteins and Vps34p that results in partial recruitment of Vps34p into the viral replication compartment in both yeast and plant cells.

To examine if Vps34p is a permanent component of the tombusvirus replicase, we shut down the formation of new tombusvirus replicase complexes by stopping ribosomal translation through adding cycloheximide to the yeast growth media [8]. Flag-affinity-purification of the tombusvirus replicase from the membrane fraction of yeast at various time-points showed the decreasing amounts of the co-purified Vps34p in the purified replicase preparations (Fig 6F, lanes 3–4 versus 2). Therefore, Vps34p seems to be released from the replicase, indicating that Vps34p is a temporarily co-opted host factor in the tombusvirus replicase complex.

### PI(3)P is enriched within the viral replication compartment

**Fig 7:**
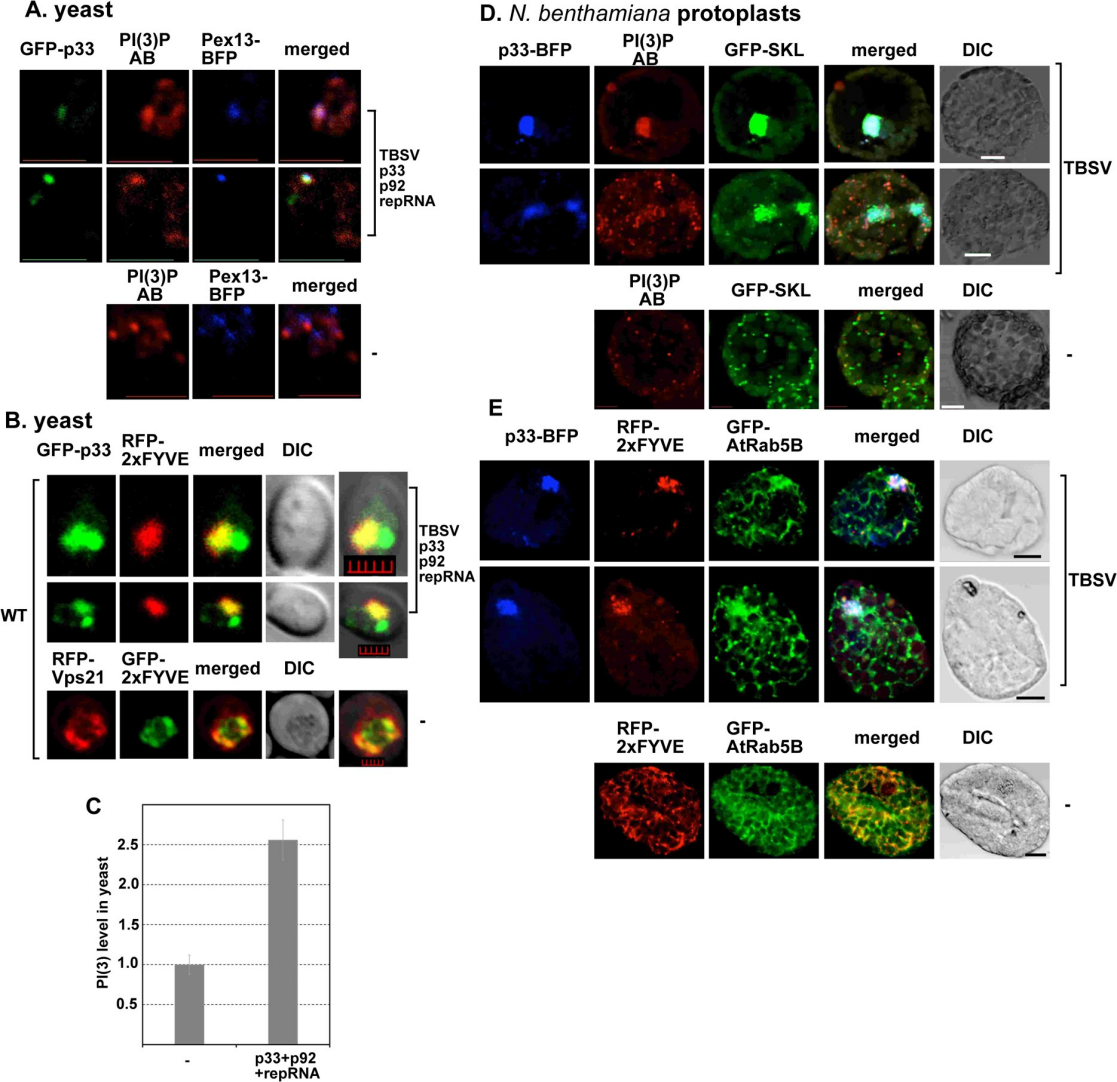
Enrichment of PI(3)P into the tombusvirus replication compartment in yeast and in *N*. *benthamiana*. (A) Confocal laser microscopy shows partial co-localization of GFP-tagged p33 replication protein with PI(3)P detected via anti-PI(3)P antibody in yeast cells replicating TBSV repRNA. Peroxisomes were detected with Pex13-BFP marker protein. The bottom panel shows confocal microscopy images of yeast lacking viral components. Scale bars represent 5 μm. (B) Confocal laser microscopy shows partial co-localization of GFP-tagged p33 replication protein with PI(3)P detected via RFP-2xFYVE protein in yeast cells replicating TBSV repRNA. See further details in panel A. Scale bars represent 2 μm. (C) Higher abundance of PI(3)P in yeast replicating TBSV repRNA. PI(3)P abundance was measured with anti-PI(3)P antibody in yeast cells with confocal microscopy and Image J software based on at least 40 cells. Standard deviation is shown. (D) Confocal laser microscopy shows partial co-localization of TBSV BFP-tagged p33 replication protein with PI(3)P detected via anti-PI(3)P antibody in *N*. *benthamiana* protoplasts infected with TBSV. The peroxisomes are marked by GFP-SKL. Expression of the above proteins from the 35S promoter was achieved after agroinfiltration into *N*. *benthamiana* leaves. Scale bars represent 10 μm. (E) Partial co-localization of p33-BFP, PI(3)P and GFP-AtRab5B protein in *N*. *benthamiana* cells infected with TBSV. PI(3)P was detected via expression of RFP-2xFYVE protein. The bottom image shows the localization of PI(3)P and GFP-AtRab5B protein in the mock-infected plant leaves. Scale bars represent 10 μm.

Based on the recruitment of Vps34p PI3K into the viral replication compartment, we assumed that the replication compartment is enriched in PI(3)P. Accordingly, detection of PI(3)P with anti-PI(3)P antibody in yeast cells replicating TBSV repRNA using confocal microscopy revealed the enrichment of PI(3)P at replication sites, which were also decorated with the peroxisomal marker (Fig 7A and S4A Fig). In the absence of TBSV components, PI(3)P was not co-localized with the peroxisomal marker (Fig 7A, bottom panel, and S4A Fig). These observations were confirmed by using a biosensor (RFP-2xFYVE), which specifically binds to PI(3)P in cells [30,57]. Expression of the RFP-tagged 2xFYVE protein domain showed partial co-localization of PI(3)P and GFP-p33 replication protein in the large replication compartment in yeast cells, whereas PI(3)P was co-localized with the Rab5-decorated (Vps21 in yeast) endosome in the absence of TBSV components (Fig 7B).

Because Vps34 level is increased in yeast and plants (Fig 3E and 3F), we also tested if PI(3)P amount is changed due to tombusvirus replication in yeast. Interestingly, we observed 2.5x fold increase in PI(3)P level in yeast replicating TBSV repRNA (Fig 7C). Thus, the increased level of Vps34p results in higher abundance of PI(3)P, which likely promotes more efficient tombusvirus replication.

Studying the PI(3)P distribution via either anti-PI(3)P antibody or RFP-tagged FYVE protein domain in *N*. *benthamiana* plant cells infected with TBSV also showed PI(3)P enrichment within the large replication compartment (Fig 7D and 7E, and S4B Fig). On the contrary, PI(3)P was not co-localized with the peroxisomal marker and it was co-localized with Rab5B (early endosome) in plant cells not infected with TBSV. Based on these results, we conclude that PI(3)P is enriched in the TBSV replication compartment in yeast and plant cells during TBSV replication.

### PI(3)P has a proviral function during tombusvirus replication

**Fig 8:**
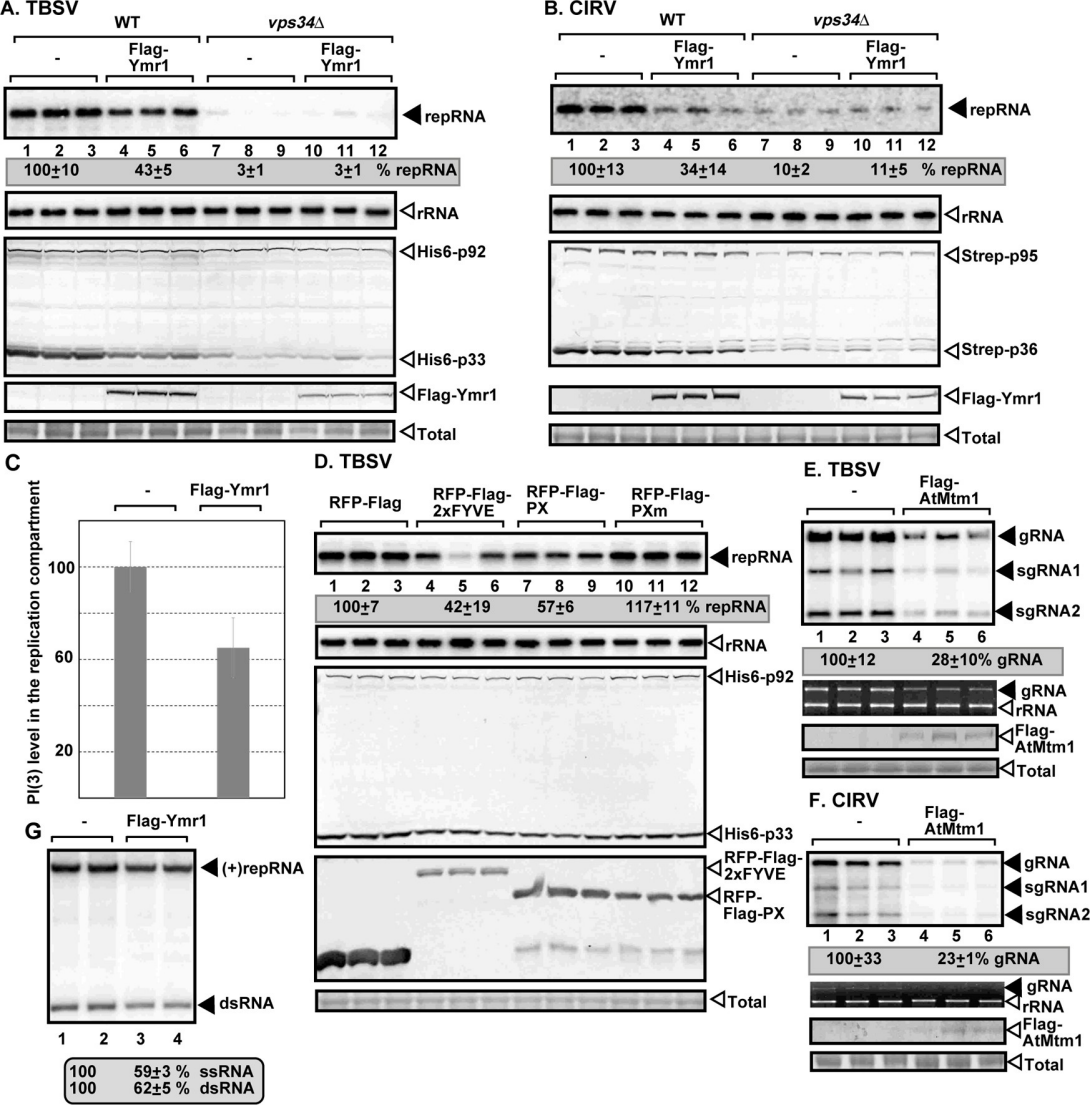
Reduction of PI(3)P inhibits tombusvirus replication in yeasts and plants. (A) Expression of yeast Ymr1p PI(3)P phosphatase, which produces PI from PI(3)P, inhibits TBSV replication in yeast. Top panel: Northern blot analysis of TBSV repRNA using a 3’ end specific probe shows reduced accumulation of repRNA in WT yeast strain expressing Ymr1p. Viral proteins His_6_-p33 and His_6_-p92^pol^ were expressed from plasmids from the *GAL1* promoter, while DI-72(+) repRNA was expressed from the *GAL10* promoter. Flag-Ymr1p was expressed from a plasmid in WT and vps34Δ yeast strains as shown. Middle panel: Northern blot with 18S ribosomal RNA specific probe was used as a loading control. Bottom images: Western blot analysis of the level of His_6_-p33 and His_6_-p92^pol^ with anti-His antibody and Flag-Ymr1p with anti-Flag antibody. (B) Reduced CIRV replication in WT yeast strain expressing of yeast Ymr1p PI(3)P phosphatase. The Strep-tagged CIRV replication proteins were detected with anti-Strep antibody. See further details in panel A. (C) Decreased abundance of PI(3)P in yeast replicating TBSV repRNA and expressing Flag-tagged Ymr1p PI(3)P phosphatase. PI(3)P abundance was measured with anti-PI(3)P antibody within the TBSV replication compartment visualized by expression of GFP-p33 in yeast cells with confocal microscopy and Image J software based on at least 40 cells. Standard deviation is shown. (D) Expression of PI(3)P-binding proteins, which sequester PI(3)P, inhibits TBSV replication in yeast. The WT PX domain and its mutant version (PXm with reduced PI(3)P binding) and the 2xFYVE domain proteins were expressed from high-copy-number plasmids. See further details in panel A. (E) Expression of Flag-tagged AtMtm1 PI(3)P phosphatase, which produces PI from PI(3)P, inhibits TBSV RNA replication in *N*. *benthamiana* plants. Top panel: Accumulation of the TBSV genomic (g)RNA in *N*. *benthamiana* plants 2 days post-inoculation was measured by Northern blot analysis. Inoculation of TBSV gRNA was done 44 h after agroinfiltration of a plasmid carrying AtMtm1 sequence. Middle panel: ribosomal RNA was used as a loading control. Bottom images: Western blot analysis of the level of Flag-AtMtm1 with anti-Flag antibody. (F) Expression of Flag-tagged AtMtm1 PI(3)P phosphatase inhibits CIRV RNA replication in *N*. *benthamiana* plants. See further details in panel E. (G) The affinity purified yeast Ymr1p PI(3)P phosphatase reduces TBSV RNA production by the tombusvirus replicase assembled *in vitro* in cell-free extract (CFE) prepared from wt yeast. Purified recombinant p33 and p92^pol^ replication proteins of TBSV and *in vitro* transcribed TBSV DI-72 (+)repRNA were added to the CFEs. Nondenaturing PAGE analysis shows the ^32^P-labeled TBSV repRNA products, including the (+)repRNA progeny and the dsRNA replication intermediate, made by the reconstituted TBSV replicase. Each experiment was repeated three times.

To test if PI(3)P has a proviral role during TBSV replication, we over-expressed the yeast Ymr1p PI(3)P phosphatase, which converts PI(3)P to PI [58]. Over-expression of Ymr1p in wt yeast reduced TBSV repRNA replication by more than 50%, whereas CIRV replication was inhibited by ~3-fold (Fig 8A and 8B), suggesting that PI(3)P is needed for both TBSV and CIRV replication. On the contrary, over-expression of Ymr1p in vps34Δ yeast, which cannot synthesize PI(3)P, does not affect TBSV or CIRV replication when compared with the accumulation level of repRNA in vps34Δ yeast (Fig 8A and 8B). We found that over-expression of Ymr1p reduced PI(3)P level by ~40% in yeast, based on immunofluorescense assay with antibody against PI(3)P (Fig 8C). Over-expression of Ymr1p in either pex3Δ or pex19Δ yeasts, which are deficient in peroxisome biogenesis and support TBSV replication via the ER membrane [26,59], led to 50–60% reduction in TBSV repRNA replication (S5 Fig). Thus, PI(3)P is required for tombusvirus replication in different subcellular locations.

We also expressed PI(3)P-binding proteins (FYVE and PX) [30], that by binding to PI(3)P, might sequester PI(3)P, thus this lipid would not be readily available for supporting TBSV replication. Indeed, these proteins inhibited TBSV replication by 40-to-60% when expressed in yeast, whereas the mutated version of PX [46,60] with reduced PI(3)P-binding did not inhibit TBSV replication in yeast (Fig 8D). We cannot exclude that the steric hindrance effect of the FYVE and PX proteins recruited to the PI(3)P-rich replication compartment also contributes to the inhibitory effect of these proteins on tombusvirus replication. Moreover, expression of the *Arabidopsis* ortholog of the yeast Ymr1, called Mtm1, also inhibited TBSV and CIRV replication in *N*. *benthamiana* plants by ~70–80% (Fig 8E and 8F).

To obtain further evidence that PI(3)P has pro-viral function during TBSV replicase reconstitution *in vitro*, we affinity-purified Flag-Ymr1p from yeast, followed by pre-incubation with CFE obtained from WT yeast. Then, we added (+)repRNA and the purified recombinant TBSV replication proteins to the CFE-based replicase reconstitution assay and measured repRNA replication. We observed a ~40% reduction in repRNA replication when the CFEs were pre-incubated with Flag-Ymr1 in comparison with pre-incubation of CFE with the buffer (Fig 8G). The accumulation of both the dsRNA replication intermediate and the newly-made (+)RNA progeny decreased in CFEs pre-incubated with Flag-Ymr1, indicating that PI(3)P function is likely required during the replicase complex assembly step *in vitro*.

Deletion of *YMR1* in yeast did not affect TBSV or CIRV replication (S6A and S6B Fig). Similarly, CFE prepared from ymr1Δ yeast supported TBSV repRNA replication close to wt level (S6C Fig), indicating that Ymr1p has no antiviral activities in yeast. All these data point at PI(3)P as a proviral host component during tombusvirus replication.

### Endomembrane/vesicle trafficking function of Vps34 PI3K is important for supporting the assembly of tombusvirus replication compartment

Vps34p forms at least four different complexes in yeast with different functions [48]. While Vps15p, the activator of Vps34p, is present in all these complexes, Atg14p is a unique component in the signaling complex regulating autophagy, whereas Vps38p is needed for the endosome trafficking function of Vps34p complex [61]. One of the two yeast Vps34p complexes regulating endosome trafficking also contains Beclin1-related *VPS30* (also called *ATG6*).

**Fig 9:**
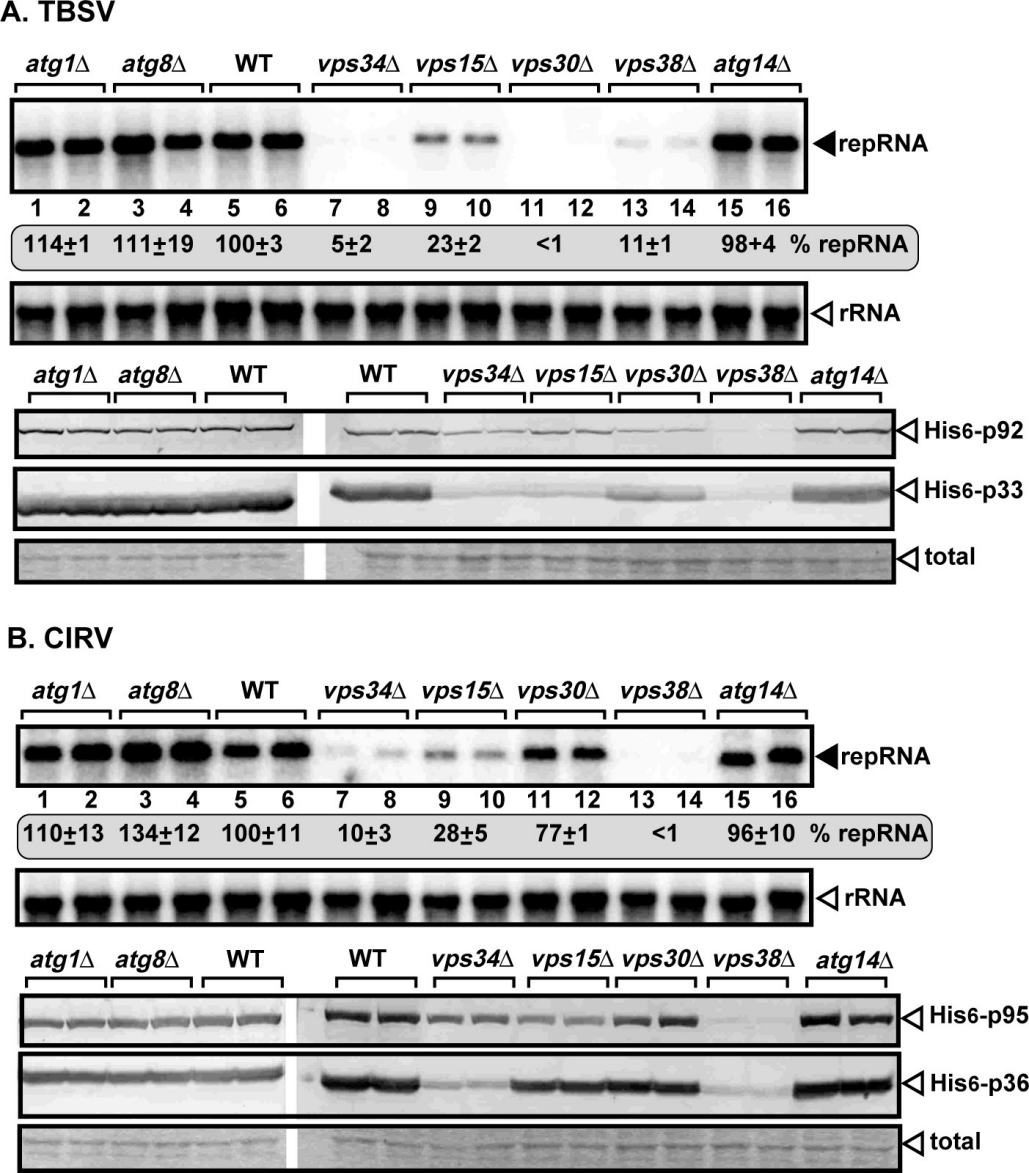
Components of the Vps34p PI3K complex are essential host factors for tombusvirus replication in yeast. (A) Deletion of selected yeast genes, which are parts of Vps34 complexes, inhibits TBSV replication in yeast. Top panel: Northern blot analysis of TBSV repRNA using a 3’ end specific probe shows the reduced accumulation of repRNA in the shown yeast strains in comparison with the wt yeast strain. Viral proteins His_6_-p33 and His_6_-p92^pol^ were expressed from plasmids from the *GAL1* promoter, while DI-72(+) repRNA was expressed from the *GAL10* promoter. Middle panel: Northern blot with 18S ribosomal RNA specific probe was used as a loading control. Bottom images: Western blot analysis of the level of His_6_-p33 and His_6_-p92^pol^ with anti-His antibody. (B) Deletion of selected yeast genes, which are parts of Vps34 complexes, inhibits CIRV replication in yeast. See further details in panel A. Each experiment was repeated three times.

To gain insights into the pro-viral functions of Vps34p and PI(3)P, we have analyzed TBSV repRNA accumulation in yeast missing proteins that form complexes with Vps34p. Deletion of *VPS15* reduced TBSV or CIRV repRNA accumulation by ~4-fold (Fig 9A and 9B, lanes 9–10). Deletion of *VPS38* component of the Vps34/Vps15p complex reduced TBSV or CIRV repRNA accumulation as much as deletion of *VPS34* (Fig 9A and 9B, lanes 13–14). The effect of deletion of Beclin1-related *VPS30* was dramatic in TBSV replication, but lesser in CIRV repRNA accumulation (Fig 9). Deletion of *VPS15*, *VPS30* (Beclin1) and *VPS38* also resulted in reduced accumulation of the p33 and p36 replication proteins of TBSV and CIRV (Fig 9A and 9B), suggesting that the pro-viral functions of Vps34p are performed by the four-component Vps34/Vps15/Vps30/Vps38 complex. Based on these data, we suggest that the vesicle trafficking function of Vps34 PI3K is important for tombusvirus replication.

Vps34p is also involved in providing PI(3)P for the initiation of autophagy, a recycling mechanism for the cells [31,33,34]. However, we found that deletion of the critical autophagy genes, such as *ATG1*, *ATG8* and *ATG14*, the latter which participate in autophagic signaling complex formation with Vps34p, did not affect TBSV or CIRV RNA accumulation in yeast under the given growth conditions (Fig 9). Similarly, deletion of additional autophagy genes *ATG5*, *ATG7* and *ATG12* did not have a major effect on TBSV replication in yeast (S6 Fig). Further experiments will be needed to analyze the roles of the autophagy genes under different conditions in tombusvirus replication. Altogether, these experiments indicated that the Vps34/Vps15/Vps30/Vps38 complex, involved in endomembrane trafficking is essential for tombusvirus replication in yeast.

### The role of Rab5 in enrichment of PI(3)P in the viral replication compartment

**Fig 10:**
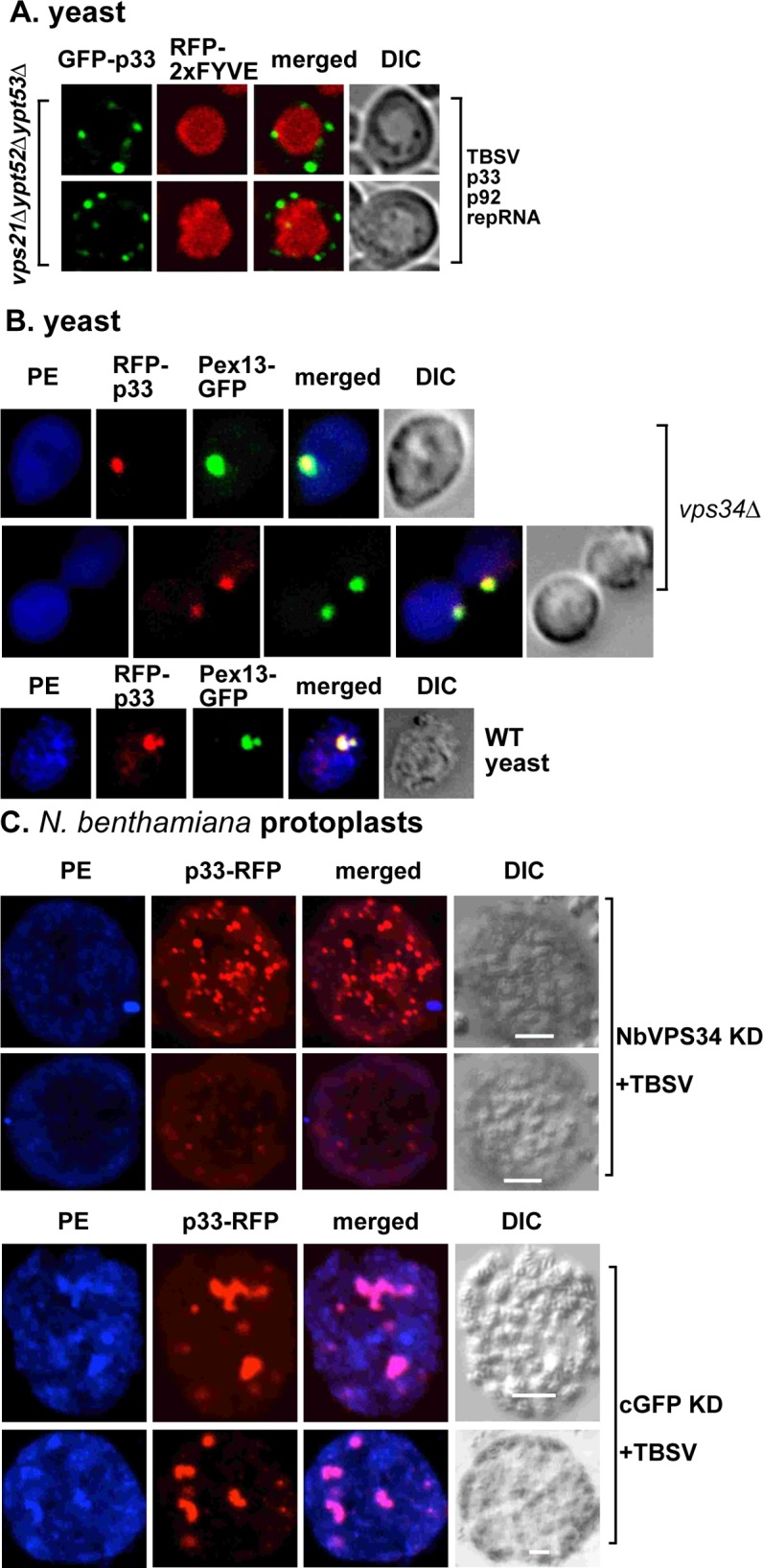
Vps34p PI3K is required for enrichment of PE within the tombusvirus replication compartment in yeast and in plants. (A) Lack of recruitment of PI(3)P into the viral replication compartment in the absence of Rab5 GTPase. PI(3)P was detected via expression of RFP-2xFYVE protein in *vps21Δypt52Δypt53Δ* yeast. (B) Top panels: Confocal laser microscopy images show the lack of enrichment of PE and its lack of co-localization with the TBSV p33/p92 replication proteins in vps34Δ yeast. PE distribution was detected by using biotinylated duramycin peptide and streptavidin conjugated with Alexa Fluor 405. DIC (differential interference contrast) images are shown on the right. Bottom panel: Enrichment of PE and its co-localization with the TBSV p33/p92 replication proteins in wt yeast is visualized by confocal microscopy. Note that yeasts also expressed p92 replication protein and the repRNA. (C) Top panels: VIGS-based knockdown of Vps34 level inhibits the enrichment of PE within the tombusvirus replication compartment *in planta*. Inoculation of TBSV gRNA was done 12 days after silencing of *VPS34* expression. VIGS was performed via agroinfiltration of tobacco rattle virus (TRV) vector carrying NbVps34 sequences. The nonspecific VIGS control was a TRV vector carrying 3’ sequences of GFP (cGFP). Protoplasts were obtained from *N*. *benthamiana* leaves, followed by detection of PE distribution with biotinylated duramycin peptide and streptavidin conjugated with Alexa Fluor 405 and confocal microscopy. Bottom images: The experiments were done as in the top panels, except TRV-cGFP was used for VIGS as a control. Each experiment was repeated three times. Scale bars represent 10, 10, 10 and 5 μm (from the top to the bottom panels).

Rab5 GTPase is important for the maturation of the early endosome and it interacts with Vps34p to perform this function [62,63]. Moreover, the p33 replication protein interacts with Rab5 that leads to the recruitment of the early endosome to the replication compartment [28]. Therefore, we tested if Rab5 facilitates PI(3)P production within the replication compartment. We found that deletion of all three Rab5 genes (vps21Δypt52Δypt53Δ) in yeast interfered with the production of PI(3)P within the replication compartment based on the lack of co-localization of RFP-2xFYVE and GFP-p33 (Fig 10A).

Previously, we found that a major pro-viral function of Rab5, whose deletion results in ~20% TBSV replication [28], is to facilitate the enrichment of PE in the viral replication compartment [28]. Vps34p and PI(3)P might be needed for this process due to their documented functions with Rab5. Accordingly, deletion of *VPS34* in yeast prevented the enrichment of PE within the replication compartment (Fig 10B and S7 Fig). Similarly, knockdown of Vps34 level in *N*. *benthamiana* resulted in poor enrichment of PE within the replication compartments, which were visibly smaller than those in the control plants (Fig 10C). Based on these data, we suggest that Vps34p is involved in facilitating the tombusvirus replication protein-driven PE enrichment within the replication compartment.

### Chemical inhibitors of PI3K inhibit the replication of several plant viruses

**Fig 11:**
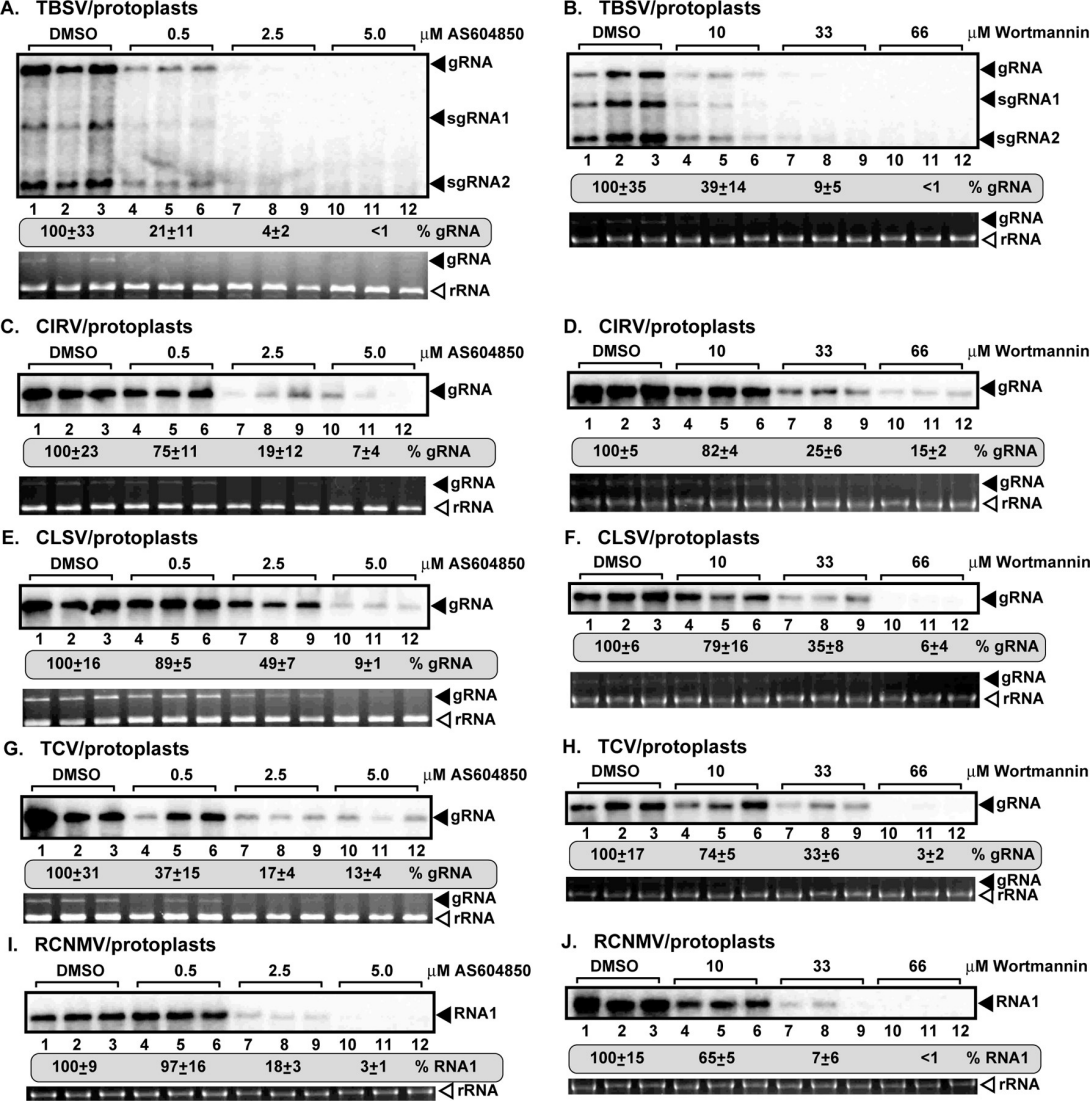
Vps34p PI3K is an essential host factor for several viruses in the Tombusviridae family. (A-B) Inhibition of TBSV replication by the PI3K inhibitor AS604850 or Wortmannin in *N*. *benthamiana* protoplasts. Northern blot analysis of TBSV gRNA using a 3’ end specific probe shows reduced accumulation of gRNA in the inhibitor-treated versus untreated protoplasts. Ethidium-bromide stained agarose gels show ribosomal RNA level as a loading control. (C-D) Northern blot analysis of CIRV gRNA using a 3’ end specific probe shows reduced accumulation of gRNA in the AS604850 or Wortmannin-treated versus untreated protoplasts. See further details in panel A. (E-F) Northern blot analysis of CLSV gRNA using a 3’ end specific probe shows the reduced accumulation of gRNA in the AS604850 or Wortmannin-treated versus untreated protoplasts. See further details in panel A. (G-H) Northern blot analysis of turnip crinkle virus (TCV) gRNA using a 3’ end specific probe shows reduced accumulation of gRNA in the AS604850 or Wortmannin-treated versus untreated protoplasts. See further details in panel A. (I-J) Northern blot analysis of RCNMV RNA1 using a 3’ end specific probe shows reduced accumulation of RNA1 in the AS604850 or Wortmannin-treated versus untreated protoplasts. See further details in panel A.

To test if Vps34p PI3K and PI(3)P play a role in the replication of other plant viruses, we treated *N*. *benthamiana* protoplasts (cell wall-free plant cells) with PI3K inhibitors, Wortmannin and AS604850, respectively [51]. We found that the replication of all five plant viruses within the Tombusviridae family, namely, TBSV, CIRV, cucumber leaf spot virus (CLSV), turnip crinkle virus (TCV), and red clover necrotic mosaic virus (RCNMV), was greatly inhibited (by up to 80–100%) by these two PI3K inhibitors (Fig 11). Thus, targeting of the cellular PI(3)P and the PI3K could lead to broad spectrum resistance against several plant viruses.

## Discussion

One of the emerging themes in RNA virus replication is that (+)RNA viruses build large replication compartments inside the cytosol of the infected cells during the infection process to sequester viral and host-components into membranous replication compartments for robust viral replication [5,9,64–66]. The replication compartment also provides protected subcellular environment for the viral dsRNA replication intermediates [5,6]. Formation of the replication compartments is driven by viral replication proteins with the help of numerous co-opted host proteins, cellular membranes and lipids [1,67–72].

In this paper, we exploited tombusviruses and yeast as simple model systems to identify new host factors involved in building the viral replication compartments. We have found a major role for the cellular Vps34p PI3K and PI(3)P phosphoinositide in the formation of tombusvirus replication compartment. The observed recruitment of Vps34p PI3K allows the production of PI(3)P within the replication compartment, which is required for robust tombusvirus replication. Accordingly, we measured little tombusvirus replication in the absence of Vps34 in yeast or when we knocked down Vps34 level in plant cells. We also observed increased expression of Vps34 and higher abundance of PI(3)P in the presence of the tombusviral replication proteins, which likely leads to more efficient tombusvirus replication.

An intriguing feature of tombusviruses is that they can utilize different subcellular organellar membranes for their replication. Accordingly, TBSV and CNV recruit Vps34p to the peroxisome/ER-based replication compartment, whereas CIRV co-opts Vps34p to the outer membranes of mitochondria. The retargeting of Vps34p into the viral replication compartment by tombusviruses seems to be based on direct interaction between the TBSV p33 or the CIRV p36 replication proteins and Vps34 PI3K. Vps34p interaction with the viral replication proteins seems to be temporal based on the decreasing amount of Vps34p in the purified replicase preparations when the assembly of new VRCs is stopped by cycloheximide. Vps34p is likely needed at the early replicase assembly/activation step since the synthesis of TBSV dsRNA replication intermediate was inhibited in CFEs prepared from vps34Δ yeast.

Vps34p participates in different complexes that perform separate functions, such as endomembrane/vesicular trafficking, autophagy, pheromone signaling and cytokinesis [31,33]. A gene deletion approach, which removed critical components of the various Vps34p complexes, indicated that all four of the known proteins in the vesicular trafficking pathway, such as Vps34p, Vps15p, Vps30p (human Beclin-1 homolog) and Vps38p, affected TBSV or CIRV replication in yeast. On the contrary, deletion of *ATG14*, which binds to Vps30 and is essential component of the autophagy and pheromone signaling pathways, had no effect on TBSV replication under the conditions used (Fig 9). Moreover, Vps34p affected PE recruitment to the site of replication (Fig 10) and PE is enriched in the endosomal membranes [28]. This further supports that the endomembrane/vesicular trafficking function of Vps34p is crucial for tombusvirus replication. Also, the findings further strengthen our previous model based on the pro-viral function of Rab5 small GTPase [28], which is required for TBSV to enrich PI(3)P within the replication compartment. Thus, tombusviruses exploit the early endosomes to usurp proteins, Vps34p and Rab5, and for enrichment of lipids, such as PE and PI(3)P, within the viral replication compartment.

The lack of early endosomal proteins Vps34p and Rab5 small GTPase had similar as well as unique effects on TBSV replication. For example, in the absence of Vps34p and Rab5, the accumulation levels of TBSV p33 and CIRV p36 replication proteins are greatly decreased, possibly due to protein degradation. Moreover, the enrichment of PE within the replication compartment depends on both co-opted host proteins. These overlapping, but not redundant, functions of Vps34p and Rab5 in tombusvirus replication might be due to the requirement of these proteins to maintain the proper endosomal network, including high PE level in the endosomal membranes, which then can be hijacked by tombusviruses. The unique function of Vps34p in comparison with Rab5 has been observed using the vps34Δ CFE-based replicase reconstitution assay, which showed reduced TBSV repRNA accumulation *in vitro*, whereas the comparable CFE assay from vps21Δypt52Δypt53Δ yeast supported as efficient TBSV repRNA replication as the CFE from WT yeast [28]. This difference indicates that the product of Vps34p PI3K, namely PI(3)P, likely plays a very important role in the VRC assembly. This model is supported by additional CFE-based assays, when addition of purified yeast Ymr1p PI(3)P phosphatase to the CFE from WT yeast reduced TBSV repRNA replication *in vitro*.

Vps34p seems to be recruited to the replication compartment to produce PI(3)P phosphoinositide, leading to highly enriched PI(3)P level in the membranes utilized for TBSV replication. The key component for tombusvirus replication is PI(3)P phosphoinositide, since Vps34p kinase mutants could not complement TBSV replication deficiency in vps34Δ yeast. And these mutants acted as dominant negative mutants by inhibiting TBSV replication in WT yeast. Moreover, expression of the PI(3)P phosphatase (yeast Ymr1p or *Arabidopsis* Mtm1) reduced TBSV and CIRV RNA accumulation. Also, sequestering PI(3)P with PI(3)P-binding proteins inhibited TBSV replication. PI(3)P is an important signaling lipid in the eukaryotic cells through interacting with many effector proteins [46,73,74], which might function in tombusvirus replication. PI(3)P level seems to affect p33 replication protein stability since p33 accumulation was reduced in vps34Δ yeast or when PI(3)P phosphatase was over-expressed.

The presented data with the proteasome inhibitor (Fig 2B) point out that the overall level of TBSV and CIRV replication is likely affected by both the reduced amount of replication proteins due to increased replication protein degradation in the absence of Vps34 and the direct contribution PI(3)P and/or Vps34 PI3K to formation of viral replication complex. The direct contribution of these host factors is also supported by *in vitro* CFE-based experiments, which demonstrated the less efficient TBSV replicase assembly in the absence of Vps34. Although the direct function of PI(3)P in tombusvirus replication is currently unknown, PI(3)P might be involved in recruitment of additional host factors, which bind to PI(3)P.

Characterization of the proviral effect of cellular PI(3)P and PI3K also opens up new approaches to target viral infections with antivirals, such as the PI3K inhibitors Wortmannin and AS604850 (Fig 11 and S1 Fig). Accordingly, we demonstrate strong inhibition of replication of tombusviruses and other related plant viruses and the unrelated NoV insect alfanodavirus. Therefore, it seems that chemical inhibition of PI3K might result in broad range protection against viruses. The NS4B replication protein of hepatitis C virus has been shown to interact with Vps34 and Rab5 [37], leading to induction of autophagy, which is beneficial for HCV replication [36].

Many (+)RNA viruses, such as picornaviruses and HCV, require PI(4)P instead of PI(3)P [70]. PI(4)P is the signature lipid of the secretory pathway, whereas PI(3)P is critical for the endosomal pathway. This difference among viruses might reflect the different membrane origins of their replication organelles. For example, poliovirus (PV) and coxsackievirus B3 hijack the Golgi and TGN and recruit PI4K to the replication organelle to produce PI(4)P *in situ* [67,75,76]. The 3C replication protein binds directly to PI(4)P and PI(4)P is required for viral RNA synthesis [75,77]. PI(4)P is also required for sterol enrichment within the replication organelles of Rhinovirus and PV [78,79].

In summary, tombusviruses take advantage of PI3K and enrich PI(3)P phosphoinositide within the viral replication compartment. This allows tombusviruses to provide optimal microenvironment for efficient VRC assembly and robust virus replication. Additional work will define if more endosomal components and PI(3)P effectors are exploited by tombusviruses for replication. Our observations of virus-mediated re-targeting of major cellular components should also be useful to understand the multiple and complex functions of cellular components and their roles in disease states.

## Materials and methods

### Yeast strains

Parental yeast strain BY4741 (*MATa his3Δ1 leu2Δ0 met15Δ0 ura3Δ0*), and deletion strains vps34Δ, ymr1Δ, atg1Δ, atg8Δ, vps15Δ, vps30Δ, vps38Δ, atg14Δ, atg5Δ, atg7Δ and atg12Δ were purchased from Open Biosystems. SC1 (*MATa his3Δ1 leu2Δ trp1Δ289 uraΔ52*) yeast strain was purchased from Invitrogen.

### Plant and yeast expression plasmids

The list of plasmids and primers are described in S1 Table.

### Analysis of viral replication in yeast and plant

To measure the effects of deletion of specific yeast genes on replication of TBSV or CIRV, yeast strains BY4741, vps34Δ, atg1Δ, atg8Δ, vps15Δ, vps30Δ, vps38Δ, atg14Δ, atg5Δ, atg7Δ, atg12Δ and ymr1Δ were separately transformed with plasmids pESC-His-p33/DI72, pYES-His-p92 and pRS315-cflag for TBSV replication or pESC-strep-p36/DI72, pYES-strep-p95 and pRS315-cflag for CIRV replication. The transformed yeast cells were pre-grown in synthetic complete medium lacking uracil, leucine and histidine (ULH^-^) supplemented with 2% glucose at 29°C for overnight. Then, tombusviral repRNA replication was induced by changing the media to synthetic complete medium (ULH^-^) supplemented with 2% galactose at 23°C for 24 h for TBSV or 30 h for CIRV. Yeast total RNA and total protein were extracted and analyzed by Northern blot and Western blot, respectively [23].

PI3K inhibitors AS604850 (Selleck Chemicals, Cat#S2681) or Wortmannin (Alfa Aesar, Cat#AAJ63983) were added to the yeast culture to test the role of Vps34 in TBSV, CIRV, NoV and CNV replication in yeast. PI3K inhibitors dissolved in DMSO were used in different concentrations (presented in the figure legends). PI3K inhibitor AS604850 was added to the cultures when virus replication was induced. Yeast strain BY4741 was transformed with plasmids pEsc-His-p33/DI72 and pYes-His-p92 for TBSV, pEsc-His-p36/DI72 and pYes-His-p95 for CIRV, pEsc-His-CNV p33/DI72 and pYes-His-CNV p92 for CNV. Tombusviral repRNA replication was induced by changing the media to synthetic complete medium lacking uracil and histidine supplemented with 2% galactose at 23°C for 24 h for TBSV and CNV, and for 30 h in case of CIRV. For measuring NoV RNA1 and RNA3 accumulation, yeast strain BY4741 was transformed with plasmid pEsc-His/Cupm/NoV/RNA1/TRSVrz. Replication was induced by adding 50 μM CuSO_4_ to the synthetic complete medium lacking histidine containing 2% glucose and then yeast was cultured for 48 h at 29°C. After the induction of viral replication and drug treatments, total RNA and total protein were isolated to evaluate virus replication level. Each experiment was repeated.

To measure the effects of sequestering cellular PI(3)P on TBSV replication, yeast strain BY4741 was transformed with plasmids pEsc-His-p33/DI72 and pYes-His-p92 and with either pRS425-Cup1-RFP or pRS425-Cup1-RFP-Flag-2xFYVE or pRS425-Cup1-RFP-Flag-PX or pRS425-Cup1-RFP-Flag-PXm. The transformed yeast cells were pre-grown in synthetic complete medium lacking uracil, leucine and histidine supplemented with 2% glucose and 50 μM CuSO_4_, followed by culturing at 29°C for 16 h. Tombusviral repRNA replication was induced by changing the media to synthetic complete medium lacking uracil, leucine and histidine supplemented with 2% galactose and 50 μM CuSO_4_ for 24 h at 23°C. Yeast total RNA and total protein were extracted and analyzed by Northern blot and Western blot, respectively.

NbVps34 gene expression was silenced using tobacco rattle virus (TRV) -based virus-induced gene silencing (VIGS) system in *N*. *benthamiana* [80,81]. After 12 d post agroinfiltration, the upper NbVps34-silenced leaves were inoculated with TBSV or CIRV sap. Plant leaf discs from inoculated leaves were collected for total RNA extraction at 2 d post inoculation (dpi) for TBSV and 2.5 dpi in case of CIRV. The control plants were treated the same way, except using TRV-cGFP. AtMtm1 was expressed in *N*. *benthamiana* by agroinfiltration. The agroinfiltrated leaves were inoculated with TBSV or CIRV sap at 44 h or 30 h post agroinfiltration. Then, the leaf discs from the inoculated leaves were collected at 2 dpi for RNA and protein detection [80].

Protoplasts were prepared from *N*. *benthamiana* callus as described previously [82]. About 5 x 10^5^ protoplasts were electroporated with 2 μg *in vitro* transcribed full-length TBSV, CIRV, CLSV, TCV genomic RNA or RCNMV RNA1. Different amounts of AS604850 or Wortmannin PI3K inhibitors (see figure legends) and DMSO (as the negative control) were added to the electroporated protoplasts, and incubated in 35 x 10 mm petri dishes in dark at room temperature for 16 hours. Total RNAs were then isolated from these protoplasts and subjected to Northern blot analysis for detection of viral RNA accumulation. Radioactive-labeled probes for Northern blotting were prepared by *in vitro* RNA synthesis using T7 RNA polymerase in the presence of [α-^32^P]UTP using PCR-amplified templates.

### Super-resolution laser microscopy analysis of Vps34 localization in yeast cells

Yeast strain BY4741 was transformed with pESC-His-p33/DI72, pYES-His-p92 and pRS315-Vps34-Flag plasmids. The transformed yeast cells were pre-grown in synthetic complete medium lacking uracil, leucine and histidine supplemented with 2% glucose medium at 29°C overnight. Tombusvirus repRNA replication was induced by changing the media to synthetic complete medium lacking uracil, leucine and histidine supplemented with 2% galactose for 21 h at 23°C. Immunofluorescence analysis was conducted as described previously [83]. Briefly, yeast cells were digested with Zymolase 20T to remove yeast cell wall, followed by simultaneous incubation with anti-p33 monoclonal mouse antibody and anti-Flag rabbit antibody (Sigma-Aldrich, Cat#F7435). Subsequently, the spheroplasts were incubated with anti-mouse secondary antibody conjugated to Alexa Flour 647 and anti-rabbit secondary antibody conjugated to ATTO488 for 1 h. Cells on the glass-bottom dishes were subjected to super-resolution microscopic observation (N-STORM Super Resolution Microscopy, Nikon).

### Confocal microscopy analysis of plant and yeast cells

To examine the subcellular localization of Vps34 in plants, *N*. *benthamiana* leaves were co-infiltrated with *Agrobacterium* carrying plasmids pGD-AtVps34-GFP or pGD-RFP-SKL together with pGD-p33-BFP (0.3 OD_600_, each). The agroinfiltrated leaves were inoculated with TBSV sap at 14 h post agroinfiltration. After 2dpi, the agroinfiltrated leaves were subjected to confocal microscopy (FV3000 confocal laser scanning microscope, Olympus) using 405 nm laser for BFP, 488 nm laser for GFP and 559 nm for RFP. Images were captured successively and merged using the FLUOVIEW software [15].

To analyze the subcellular localization of Vps34 in yeast cells, pYes-His-p92, pEsc-GFP-p33/DI72 and pRS315-Vps34-RFP were transformed into BY4741 yeast. The transformed yeast cells were pre-grown in synthetic complete medium lacking uracil, leucine and histidine supplemented with 2% glucose at 29°C overnight. Tombusviral repRNA replication was induced by changing the media to synthetic complete medium lacking uracil, leucine and histidine supplemented with 2% galactose for 21 h at 23°C. Yeast cells were subjected to confocal microscopy analysis using 488 nm laser for GFP and 559 nm for RFP in an Olympus FV1200 confocal laser scanning microscope. The co-localization between p33 and Vps34 in yeast was quantitated from the p33 point of view using the FLUOVIEW software.

To test whether Vps34 localizes to the peroxisome upon virus replication in yeast cells, pYes-His-p92, pEsc-His-p33/DI72, pRS314-Pex13-GFP and pRS315-Vps34-RFP were transformed into SC1 yeast. The transformed yeast cells were pre-grown in synthetic complete medium lacking uracil, leucine, tryptophan and histidine supplemented with 2% glucose medium at 29°C overnight. Tombusviral repRNA replication was induced by changing the media to synthetic complete medium lacking uracil, leucine, tryptophan and histidine supplemented with 2% galactose for 21 h at 23°C. Yeast cells were subjected to confocal microscopy analysis using 488 nm laser for GFP and 559 nm for RFP in an Olympus FV1200 confocal laser scanning microscope.

To observe PI(3)P localization upon virus replication, plant protoplasts or yeast spheroplasts were isolated and treated (as described above), then subjected to immunofluorescence analysis. The permeabilized cells were incubated with purified anti-PI3P mouse antibody (Echelon Biosciences Inc. Cat#Z-P003), and then incubated with anti-mouse secondary antibody conjugated with Alexa Fluor 568 (Thermo Fisher Scientific, Cat#A11031). The cells were imaged with Olympus FV1200 confocal laser scanning microscope. The intensity profiles of the images were processed and exported using the FLUOVIEW software [15].

PI(3)P biosensor was used to determine PI(3)P localization upon virus replication. Yeast strains BY4741 and *vps21Δypt52Δypt53Δ* were transformed with pEsc-GFP-p33/DI72, pYes-His-p92 and pRS315-RFP-2xFYVE plasmids. The transformed yeast cells were pre-grown in synthetic complete medium lacking uracil, leucine and histidine supplemented with 2% glucose, followed by culturing at 29°C overnight. Tombusviral repRNA replication was induced by changing the media to synthetic complete medium lacking uracil, leucine and histidine supplemented with 2% galactose for 21 h at 23°C. Yeast cells were subjected to confocal microscopic analysis using 488-nm laser for GFP and 559-nm for RFP in an Olympus FV1200 confocal laser scanning microscope.

To identify interaction between Vps34 and TBSV p33 or CIRV p36 replication proteins *in vivo*, bimolecular fluorescence complementation (BiFC) assay was conducted [13]. The plasmids pGD-p33-cYFP or pGD-p36-cYFP, pGD-nYFP-AtVps34, pGD-nYFP-MBP, pGD-RFP-SKL and pGD-CoxIV-RFP were transformed to *Agrobacterium* strain C58C1. The obtained *Agrobacterium* transformants were co-agroinfiltrated (0.3 OD_600_, each) into the leaves of four weeks-old *N*. *benthamiana* plants. Agroinfiltrated leaves were inoculated with TBSV or CIRV 14 h after agroinfiltration. Agroinfiltrated leaves were subjected to confocal laser microscopy at 48 h post infiltration.

Additional methods used are described in S1 Text.

## Supporting information

S1 Fig Vps34 PI3K is an essential host factor for several viruses replicating in yeast. (A) Inhibition of CIRV replication by the PI3K inhibitor AS604850 in yeast. Northern blot analysis of CIRV repRNA using a 3’ end specific probe shows the reduced accumulation of repRNA in the inhibitor-treated versus DMSO-treated yeast cells. Ethidium-bromide stained agarose gel shows ribosomal RNA levels as loading control. (B) Northern blot analysis of repRNA accumulation in yeast supported by the p33 and p92 replication proteins of CNV using a 3’ end specific probe. See further details in panel A. (C) Northern blot analysis of nodamuravirus (NoV) RNA1 and RNA3 (subgenomic RNA) accumulation in yeast using a 3’ end specific probe. See further details in panel A.
(PDF)

S2 Fig Vps34 does not localize in the peroxisomes in mock-inoculated *N*. *benthamiana*. Confocal microscopy images show the localization of AtVps34-GFP and RFP-SKL peroxisomal matrix marker protein. Expression of the above proteins from the 35S promoter was done after co-agroinfiltration into *N*. *benthamiana* leaves. Scale bars represent 5 μm. See further details in Fig 5.
(PDF)

S3 Fig Interaction between p33 replication protein and Rab5 in vps34Δ yeast. Co-purification of 6xHis-tagged Vps21 (Rab5 ortholog) with the Flag-p33 replication protein from subcellular membranes of yeast. Top two panels: Western blot analysis of co-purified Vps21 (lanes 2 and 4) with Flag-affinity purified Flag-p33. Vps21 was detected with anti-His antibody, whereas p33 was detected with anti-FLAG antibody as shown. Bottom two panels: Western blot of total Vps21 and p33 in the total yeast extracts.
(PDF)

S4 Fig Intensity profile of enrichment of cellular PI(3)P in peroxisomes containing the p33 replication protein in yeast and in *N*. *benthamiana*. (A) Intensity profile of partial co-localization of GFP-tagged p33 replication protein (green line) with PI(3)P (red line) detected with anti-PI(3)P antibody in yeast cells replicating TBSV repRNA. Peroxisomes were detected with Pex13-BFP marker protein (blue line). The right panel shows the intensity profile of yeast without viral components. Note that the same yeast cells are shown here as in Fig 7A. (B) Intensity profile of partial co-localization of BFP-tagged p33 replication protein (blue line) with PI(3)P (red line) detected with anti-PI(3)P antibody in *N*. *benthamiana* protoplasts infected with TBSV. Peroxisomes were detected with GFP-SKL marker protein (green line). The right panel shows the intensity profile of protoplast from mock-infected plant leaves. Note that the same plant cells are shown here as in Fig 7D.
(PDF)

S5 Fig Reduction of PI(3)P level inhibits endoplasmic reticulum-based tombusvirus replication in yeast lacking peroxisomes. (A) Expression of yeast Ymr1p PI(3)P phosphatase, which dephosphorylates PI(3)P to PI, inhibits TBSV replication in yeast missing *PEX3* gene. Top panel: Northern blot analysis of TBSV repRNA using a 3’ end specific probe shows the reduced accumulation of repRNA in *pex3Δ* yeast strain expressing Ymr1p. Viral proteins His_6_-p33 and His_6_-p92^pol^ were expressed from plasmids from the *GAL1* promoter, while DI-72(+) repRNA was expressed from a plasmid from the *GAL10* promoter. Middle panel: Northern blot with 18S ribosomal RNA-specific probe was used as a loading control. Bottom images: Western blot analysis of the accumulation level of His_6_-p33 and His_6_-p92^pol^ with anti-His antibody and Flag-Ymr1p with anti-Flag antibody. (B) Expression of yeast Ymr1p PI(3)P phosphatase decreases TBSV replication in yeast missing *PEX19* gene. See further details in panel A. Note: TBSV replication switches from peroxisome to the endoplasmic reticulum (ER) in yeast in the absence of either *PEX3* or *PEX19* genes.
(PDF)

S6 Fig Deletion of Ymr1 and autophagy-related genes (ATGs) has little affect on tombusvirus replication in yeast. (A) Deletion of selected yeast genes shows minor affect on TBSV replication in yeast. Top panel: Northern blot analysis of TBSV repRNA using a 3’ end specific probe shows the accumulation of repRNA in the given yeast strains in comparison with the wt yeast strain (BY4741). Viral proteins His_6_-p33 and His_6_-p92^pol^ were expressed from plasmids from the *GAL1* promoter, while DI-72(+) repRNA was expressed from a plasmid from the *GAL10* promoter. Middle panel: Northern blot with 18S ribosomal RNA specific probe was used as a loading control. Bottom images: Western blot analysis of the level of His_6_-p33 and His_6_-p92^pol^ with anti-His antibody. (B) Deletion of selected yeast genes does not alter CIRV replication level in yeast. See further details in panel A. Each experiments was repeated three times. (C) Slightly reduced activities of the tombusvirus replicase assembled *in vitro* in CFEs prepared from from *ymr1Δ* in comparison with those from wt yeasts. Purified recombinant p33 and p92^pol^ replication proteins of TBSV and *in vitro* transcribed TBSV DI-72(+) repRNA were used to program the CFEs. Nondenaturing PAGE analysis shows the ^32^P-labeled TBSV repRNA products, including the (+)repRNA progeny and the dsRNA replication intermediate, produced by the reconstituted TBSV replicase *in vitro*.
(PDF)

S7 Fig Vps34p PI3K is required for enrichment of cellular PE phospholipid in peroxisomes decorated with the p33 and p92 replication proteins in yeast. Intensity profiles show the lack of enrichment of PE and its lack of co-localization of RFP-tagged p33/p92 replication proteins (red line) with PE (blue line) detected with biotinylated duramycin peptide and streptavidin conjugated with Alexa Fluor 405 in *vps34Δ* yeast in comparison with the wt yeast strain replicating TBSV repRNA. Peroxisomes were detected with Pex13-GFP marker protein (green line). Note that the same yeast cells are shown here as in Fig 10B.
(PDF)

S1 Text Experimental procedures. (PDF)

S1 Table List of primers and constructs used in this study. (PDF)
