# Supplementary figures and images for: Recruitment of Vps34 PI3K and enrichment of PI3P phosphoinositide in the viral replication compartment is crucial for replication of a positive-strand RNA virus
Source: PLoS Pathog. 2019 Jan 9;15(1):e1007530. doi: 10.1371/journal.ppat.1007530 (PMC6342326; doi:10.1371/journal.ppat.1007530)

S1 FIGURE

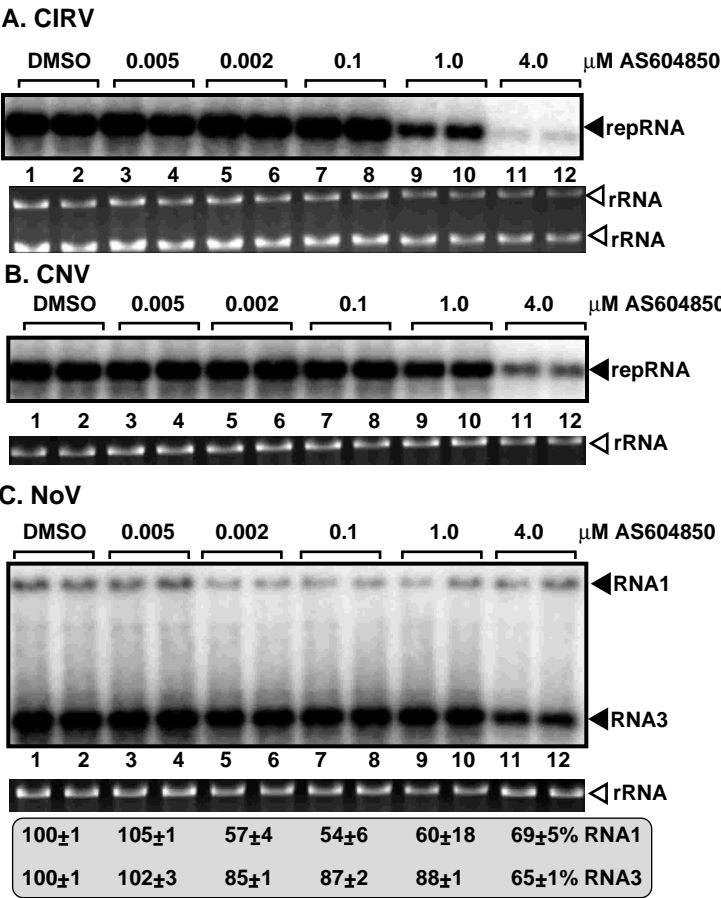

Supplement: S1 Fig — (A) Inhibition of CIRV replication by the PI3K inhibitor AS604850 in yeast. Northern blot analysis of CIRV repRNA using a 3’ end specific probe shows the reduced accumulation of repRNA in the inhibitor-treated versus DMSO-treated yeast cells. Ethidium-bromide stained agarose gel shows ribosomal RNA levels as loading control. (B) Northern blot analysis of repRNA accumulation in yeast supported by the p33 and p92 replication proteins of CNV using a 3’ end specific probe. See further details in panel A. (C) Northern blot analysis of nodamuravirus (NoV) RNA1 and RNA3 (subgenomic RNA) accumulation in yeast using a 3’ end specific probe. See further details in panel A. (PDF) [file ppat.1007530.s001.pdf]

## S2 FIGURE

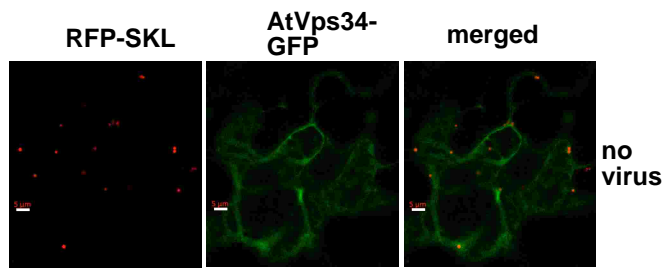

Supplement: S2 Fig — Confocal microscopy images show the localization of AtVps34-GFP and RFP-SKL peroxisomal matrix marker protein. Expression of the above proteins from the 35S promoter was done after co-agroinfiltration into N. benthamiana leaves. Scale bars represent 5 μm. See further details in Fig 5. (PDF) [file ppat.1007530.s002.pdf]

**S3 FIGURE**

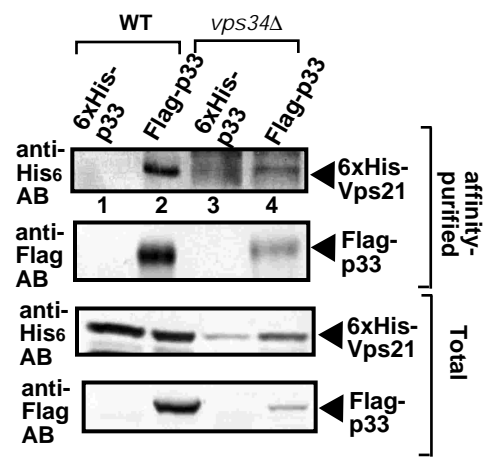

Supplement: S3 Fig — Co-purification of 6xHis-tagged Vps21 (Rab5 ortholog) with the Flag-p33 replication protein from subcellular membranes of yeast. Top two panels: Western blot analysis of co-purified Vps21 (lanes 2 and 4) with Flag-affinity purified Flag-p33. Vps21 was detected with anti-His antibody, whereas p33 was detected with anti-FLAG antibody as shown. Bottom two panels: Western blot of total Vps21 and p33 in the total yeast extracts. (PDF) [file ppat.1007530.s003.pdf]

S5 FIGURE

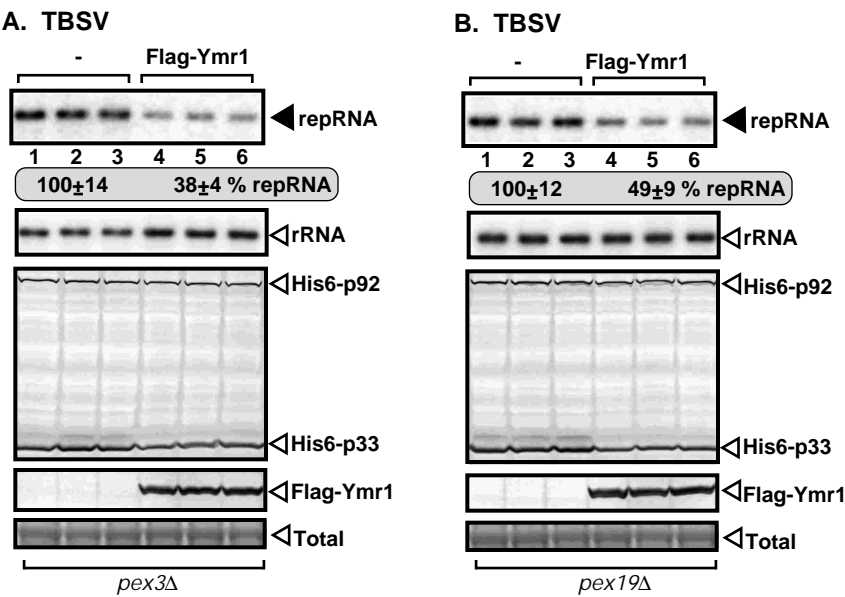

Supplement: S5 Fig — (A) Expression of yeast Ymr1p PI(3)P phosphatase, which dephosphorylates PI(3)P to PI, inhibits TBSV replication in yeast missing PEX3 gene. Top panel: Northern blot analysis of TBSV repRNA using a 3’ end specific probe shows the reduced accumulation of repRNA in pex3Δ yeast strain expressing Ymr1p. Viral proteins His6-p33 and His6-p92pol were expressed from plasmids from the GAL1 promoter, while DI-72(+) repRNA was expressed from a plasmid from the GAL10 promoter. Middle panel: Northern blot with 18S ribosomal RNA-specific probe was used as a loading control. Bottom images: Western blot analysis of the accumulation level of His6-p33 and His6-p92pol with anti-His antibody and Flag-Ymr1p with anti-Flag antibody. (B) Expression of yeast Ymr1p PI(3)P phosphatase decreases TBSV replication in yeast missing PEX19 gene. See further details in panel A. Note: TBSV replication switches from peroxisome to the endoplasmic reticulum (ER) in yeast in the absence of either PEX3 or PEX19 genes. (PDF) [file ppat.1007530.s005.pdf]

S6 FIGURE

A. TBSV

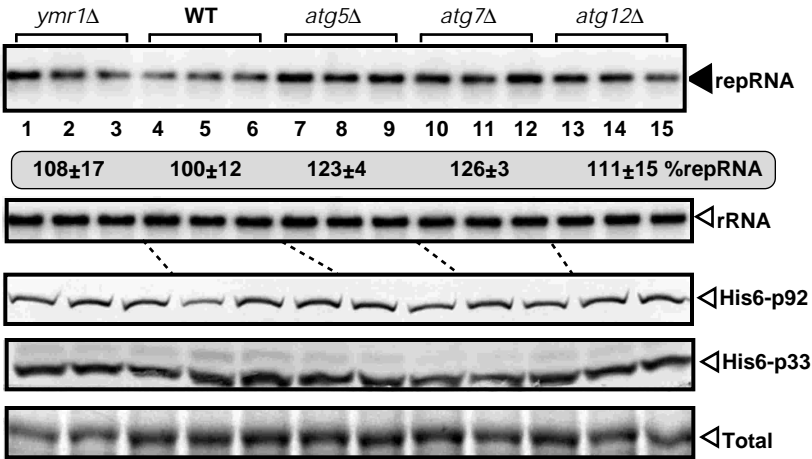

B. CIRV

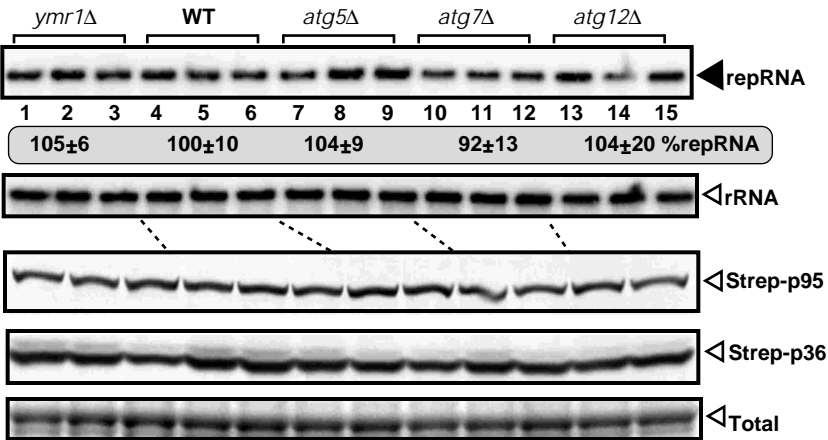

C. CFE replication assay:

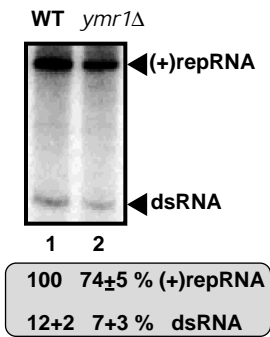

Supplement: S6 Fig — (A) Deletion of selected yeast genes shows minor affect on TBSV replication in yeast. Top panel: Northern blot analysis of TBSV repRNA using a 3’ end specific probe shows the accumulation of repRNA in the given yeast strains in comparison with the wt yeast strain (BY4741). Viral proteins His6-p33 and His6-p92pol were expressed from plasmids from the GAL1 promoter, while DI-72(+) repRNA was expressed from a plasmid from the GAL10 promoter. Middle panel: Northern blot with 18S ribosomal RNA specific probe was used as a loading control. Bottom images: Western blot analysis of the level of His6-p33 and His6-p92pol with anti-His antibody. (B) Deletion of selected yeast genes does not alter CIRV replication level in yeast. See further details in panel A. Each experiments was repeated three times. (C) Slightly reduced activities of the tombusvirus replicase assembled in vitro in CFEs prepared from from ymr1Δ in comparison with those from wt yeasts. Purified recombinant p33 and p92pol replication proteins of TBSV and in vitro transcribed TBSV DI-72(+) repRNA were used to program the CFEs. Nondenaturing PAGE analysis shows the 32P-labeled TBSV repRNA products, including the (+)repRNA progeny and the dsRNA replication intermediate, produced by the reconstituted TBSV replicase in vitro. (PDF) [file ppat.1007530.s006.pdf]

S7 FIGURE

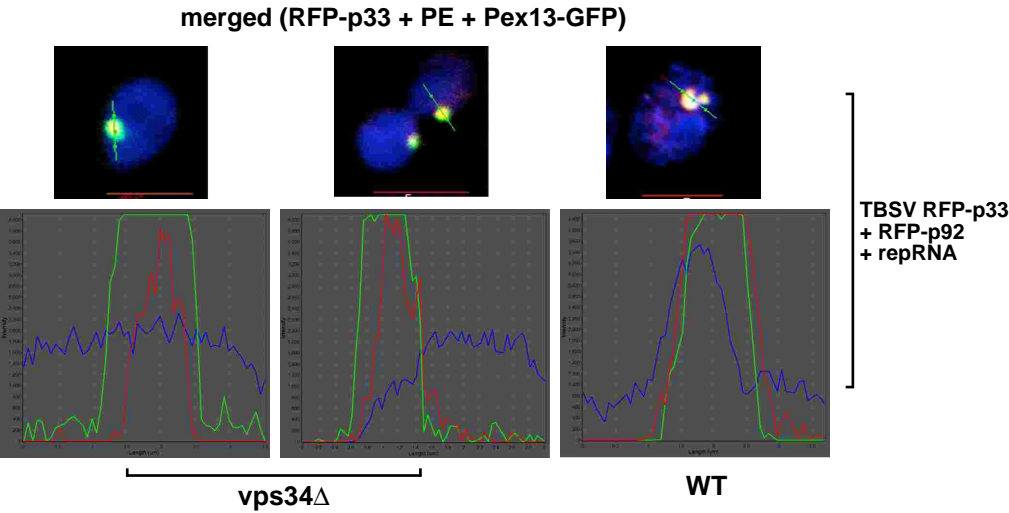

Supplement: S7 Fig — Intensity profiles show the lack of enrichment of PE and its lack of co-localization of RFP-tagged p33/p92 replication proteins (red line) with PE (blue line) detected with biotinylated duramycin peptide and streptavidin conjugated with Alexa Fluor 405 in vps34Δ yeast in comparison with the wt yeast strain replicating TBSV repRNA. Peroxisomes were detected with Pex13-GFP marker protein (green line). Note that the same yeast cells are shown here as in Fig 10B. (PDF) [file ppat.1007530.s007.pdf]
