# Supplementary material for: Recruitment of Vps34 PI3K and enrichment of PI3P phosphoinositide in the viral replication compartment is crucial for replication of a positive-strand RNA virus
Source: PLoS Pathog. 2019 Jan 9;15(1):e1007530. doi: 10.1371/journal.ppat.1007530 (PMC6342326; doi:10.1371/journal.ppat.1007530)
Supplement: S4 Fig — (A) Intensity profile of partial co-localization of GFP-tagged p33 replication protein (green line) with PI(3)P (red line) detected with anti-PI(3)P antibody in yeast cells replicating TBSV repRNA. Peroxisomes were detected with Pex13-BFP marker protein (blue line). The right panel shows the intensity profile of yeast without viral components. Note that the same yeast cells are shown here as in Fig 7A. (B) Intensity profile of partial co-localization of BFP-tagged p33 replication protein (blue line) with PI(3)P (red line) detected with anti-PI(3)P antibody in N. benthamiana protoplasts infected with TBSV. Peroxisomes were detected with GFP-SKL marker protein (green line). The right panel shows the intensity profile of protoplast from mock-infected plant leaves. Note that the same plant cells are shown here as in Fig 7D. (PDF) [file ppat.1007530.s004.pdf]

S4 FIGURE

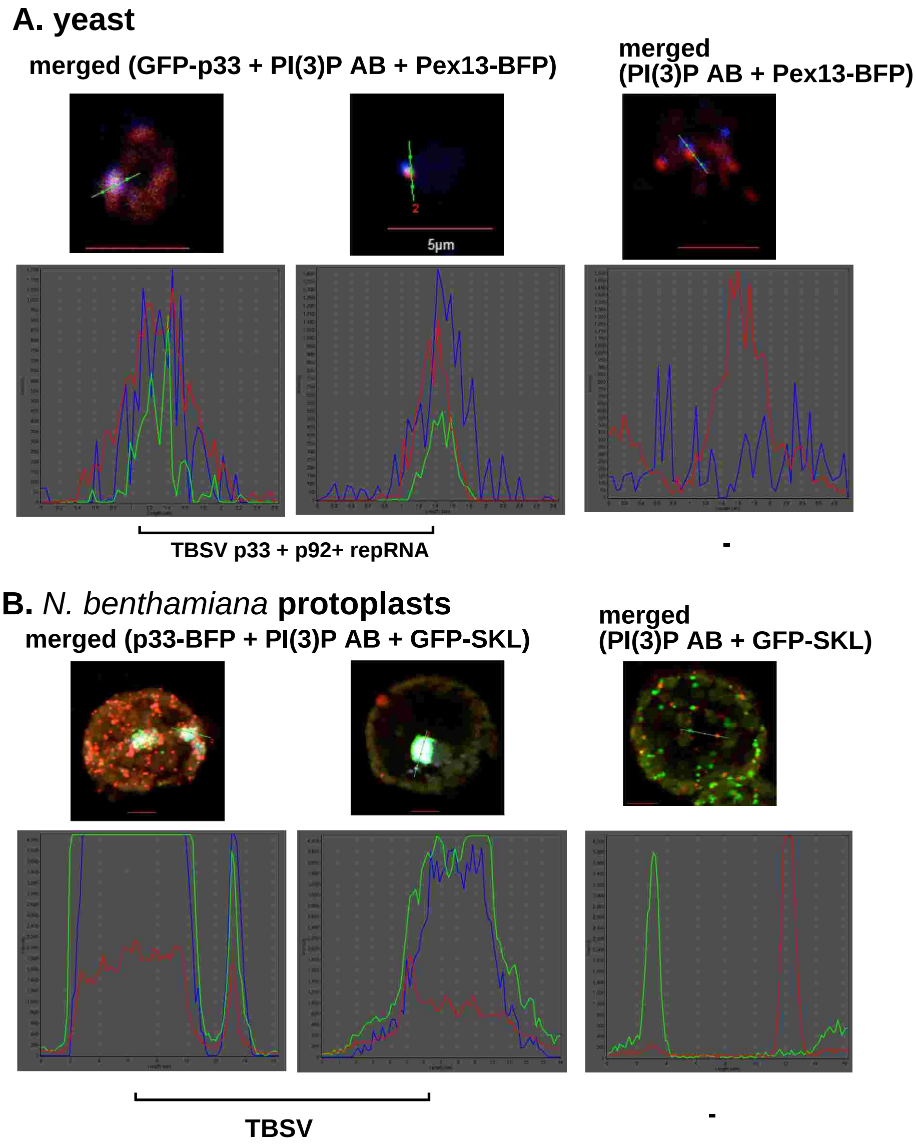
A. yeast
merged (GFP-p33 + PI(3)P AB + Pex13-BFP)
merged
(PI(3)P AB + Pex13-BFP)
2
5μm
TBSV p33 + p92+ repRNA
-
B. N. benthamiana protoplasts
merged (p33-BFP + PI(3)P AB + GFP-SKL)
merged
(PI(3)P AB + GFP-SKL)
TBSV
-
