# Supplementary material for: Recruitment of Vps34 PI3K and enrichment of PI3P phosphoinositide in the viral replication compartment is crucial for replication of a positive-strand RNA virus
Source: PLoS Pathog. 2019 Jan 9;15(1):e1007530. doi: 10.1371/journal.ppat.1007530 (PMC6342326; doi:10.1371/journal.ppat.1007530)
Supplement: S1 Text — (PDF) [file ppat.1007530.s008.pdf]

S1 Text. Experimental procedures

Protein interaction between Vps34 and p33 by co-purification and pull down assays

To test the interaction between p33 and Vps34 by co-purification assay in plant, agrobacterium (0.3 OD_{600}, each) harboring pGD-AtVps34-Flag was infiltrated into plant leaves, followed by TBSV sap inoculation 12 h post agroinfiltration. Total protein was isolated from the leaves 2 dpi. Membrane fraction was solubilized with 1% Triton X-100 followed by purification of AtVps34-Flag with anti-FLAG M2 agarose (Sigma-Aldrich, Cat#A2220). The co-purified protein was detected by anti-p33 rabbit antibody [1].

To test the interaction between p33 and Vps34 in yeast, pRS315-Vps34-Flag was transformed into yeasts with pYes-His-p92 and pEsc-His-p33/DI72. The transformed yeast cells were pre-grown in synthetic complete medium lacking uracil, leucine and histidine supplemented with 2% glucose at 29°C overnight. Tombusviral repRNA replication was induced by changing the media to synthetic complete medium lacking uracil, leucine and histidine supplemented with 2% galactose for 24 h at 23°C. Reciprocal co-purification assay was conducted by transforming BY4741 yeast with pEsc-Vps34-3xHA with pGAD-Cup1-Flag-p92 and pGBK-Cup1-Flag-p33-Gal1-DI72. Yeast transformed with pGAD-Cup1-His-p92 and pGBK-Cup1-His-p33-Gal1-DI72 was used as the negative control. The transformed yeast cells were pre-grown in synthetic complete medium lacking uracil, leucine and histidine supplemented with 2% glucose and 100 μM BCS at 29°C overnight. This was followed by transferring the yeasts to synthetic complete medium lacking uracil, leucine and histidine supplemented with 2% galactose and 100 μM BCS at 29°C for 24 h. Tombusviral repRNA replication was induced by changing the media to synthetic complete medium lacking uracil, leucine and histidine supplemented with 2% galactose and 50 μM $CuSO_4$ for 5 h at 23°C. Cross-linking of yeast cells was conducted by suspending

yeasts with 1 x PBS buffer containing 1% formaldehyde for 1 h on ice. Then, glycine (to 0.1 M) was added to quench the formaldehyde and the yeasts were collected by centrifugation [2]. Yeast cells were broken with glass beads, followed by collection of membrane fraction with centrifugation and solubilization with 1% Triton X-100. The samples were purified on anti-FLAG M2 agarose (Sigma-Aldrich, Cat#A2220). The co-purified proteins were detected by Western blotting using anti-His or anti-HA antibody.

To determine whether the interaction between p33 and Vps34 is direct or indirect, MBP-p33 was obtained from *E. coli* (BL21 DE3 Codon Plus cells), whereas proteins 2xFlag-Vps34, 2xFlag-Vps34[N736K], 2xFlag-Vps34[D749E] were purified from BY4741 yeast, whereas proteins GST-Vps34 and GST-Vps34[D749E] were purified from *E.coli* (BL21 DE3 Codon Plus cells). First, we incubated 2 μg MBP-p33 with Amylose resin (New England Biolabs, Cat#E8021L) at 4°C for 2 h. The same amount of MBP protein was used as the control. The unbound proteins were removed by repeated washing, followed by adding 1 μg 2xFlag-Vps34, 2xFlag-Vps34[N736K] or 2xFlag-Vps34[D749E] or GST-Vps34 or GST-Vps34[D749E], respectively, onto Amylose resin at 4°C for 4 h. The bound proteins were eluted with maltose [2].

Analyses of phosphatidylethanolamine (PE) enrichment within the viral replication compartment in plants and yeast cells

For PE enrichment analysis in plant cells, TRV-based NbVps34-silenced plant leaves were agroinfiltrated with agrobacterium (0.3 OD_{600}) harboring pGD-p33-RFP plasmid. The infiltrated leaves were inoculated with TBSV sap at 12 h post agroinfiltration. Protoplasts were isolated 48 h post virus inoculation for PE visualization, the method for PE-labeling has been

described previously [3]. Mesophyll protoplasts were prepared from fresh leaves of NbVps34-silenced and control plants by digesting the leaf strips with enzyme solution containing 1.5% (wt/vol) Cellulase R10 (Yakult Pharmaceutical Ind. Co., Ltd., Japan) and 0.4% (wt/vol) Macerozyme R10 (Yakult Pharmaceutical Ind. Co., Ltd., Japan), and the obtained protoplasts were used immediately for PE labelling.

For PE enrichment analysis in yeast cells, we used wild-type BY4741 and vps34Δ yeasts transformed with pYes-RFP-p92, pEsc-RFP-p33/DI72 and pRS315-Pex13-GFP plasmids. After virus replication was induced, the spheroplasts were isolated by removing yeast cell wall for PE-labeling [3].

***In vitro* cell-free extract-based replicase reconstitution assay**

To test the role of Vps34 in TBSV replication *in vitro*, cell-free extracts (CFEs) were prepared from untransformed BY4741, vps34Δ and ymr1Δ yeast strains as described [4,5]. Reaction mixture for the *in vitro* TBSV replication contained 2 μl of CFE, 0.15 μg (+) repRNA (DI-72) RNA, 400 ng affinity-purified MBP-p33 (obtained from *E. coli*), 400 ng affinity-purified MBP-p92^{pol} (obtained from *E. coli*) in 20 μl total volume. The reactions were performed for 3 h at 25°C, followed by nondenaturing polyacrylamide gel analysis [6].

To test the effect of Ymr1p PI(3)P phosphatase on TBSV replication *in vitro*, CFEs were prepared from untransformed BY4741. Then, we added purified Ymr1 protein (1 pmol) to the CFEs at 25°C for 50 min to reduce the PI(3)P level in CFEs. The obtained pre-treated (2 μl) of CFEs were mixed with 0.15 μg (+) DI-72 RNA, 400 ng affinity-purified MBP-p33, and 400 ng affinity-purified MBP-p92^{pol} in 20 μl total volume, followed by incubation at 25°C for 3 h. The

obtained ^{32}P-labeled viral repRNA products were analyzed via nondenaturing polyacrylamide gels.

To determine the effect of PI3K inhibitor Wortmannin on TBSV replication in vitro, CFEs were prepared from yeast treated with 33 μM Wortmannin at 30 °C for 2 h, then 37°C for 15 min. Then, we added 2 μl of Wortmannin-treated CFE or DMSO-treated control CFE, 0.15 μg (+) DI-72 RNA, 400 ng affinity-purified MBP-p33, 400 ng affinity-purified MBP-p92^{pol} to the reaction mixture of 20 μl total volume, and performed the CFE assay as above at 25°C for 3 h.

Protein analysis by Western blotting

Yeast total proteins were isolated by the NaOH method as described previously [7]. The total protein samples were analyzed by SDS-PAGE and Western blotting with anti-His, anti-HA and anti-Flag antibodies, followed by alkaline phosphatase-conjugated anti-mouse secondary antibody (Sigma-Aldrich, Cat#5153), using NBT/BCIP solution to develop the PVDF membranes. For detection of Flag-AtMtm1 expression in *N. benthamiana* plants, total protein extracts were prepared as described previously [8]. For Western blots we used anti-Flag rabbit antibody. To detect the accumulation level of Vps34 mutants and test the interaction between p33 and Vps34 in yeast, anti-mouse secondary antibody conjugated with horseradish peroxidase (GE Healthcare, Cat#NA931) were incubated with the membrane after anti-Flag antibody or anti-HA antibody incubation, using chemiluminescent detection reagent (GE Healthcare, Cat#RPN2232) to develop the membrane.

Inhibition of proteasome with MG132 treatment.

To check tombusvirus p33 replication protein stability upon Vps34 PI3K depletion, yeast strain BY4741 was transformed with pEsc-His-p33/DI72, pYes-His-p92 and pRS315-EV. The transformed yeast cells were pre-grown in synthetic complete medium lacking uracil, leucine and histidine, supplemented with 2% glucose medium, culture at 29°C overnight, tombusviral repRNA replication was induced by changing the media to synthetic complete medium lacking uracil, leucine and histidine with 2% galactose and 4 μM AS604850 or DMSO for 16 h at 23°C. We then collected the yeast pellet, and yeast spheroplasts were obtained with Zymolyase 20T treatment. After two washes, added 100 μg /ml Cycloheximide and 80 μM MG132 (Enzo Life Sciences, Cat#BML-PI102) for 2 h at 23°C, extracted the total protein [9]. Total protein was analyzed by Western blotting using anti-His or anti-PGK1 (internal control, Invitrogen Cat#A-6457) antibodies.

References:

1. Scholthof KB, Scholthof HB, Jackson AO (1995) The tomato bushy stunt virus replicase proteins are coordinately expressed and membrane associated. Virology 208: 365-369.
2. Chuang C, Prasanth KR, Nagy PD (2017) The Glycolytic Pyruvate Kinase Is Recruited Directly into the Viral Replicase Complex to Generate ATP for RNA Synthesis. Cell Host Microbe 22: 639-652 e637.
3. Xu K, Nagy PD (2015) RNA virus replication depends on enrichment of phosphatidylethanolamine at replication sites in subcellular membranes. Proc Natl Acad Sci U S A 112: E1782-E1791.
4. Pogany J, Stork J, Li Z, Nagy PD (2008) In vitro assembly of the Tomato bushy stunt virus replicase requires the host Heat shock protein 70. Proc Natl Acad Sci U S A 105: 19956-19961.
5. Pogany J, Nagy PD (2008) Authentic replication and recombination of Tomato bushy stunt virus RNA in a cell-free extract from yeast. J Virol 82: 5967-5980.
6. Kovalev N, Pogany J, Nagy PD (2012) A Co-Opted DEAD-Box RNA Helicase Enhances Tombusvirus Plus-Strand Synthesis. PLoS Pathog 8: e1002537.
7. Panavas T, Nagy PD (2003) Yeast as a model host to study replication and recombination of defective interfering RNA of Tomato bushy stunt virus. Virology 314: 315-325.

8. Feng Z, Chen X, Bao Y, Dong J, Zhang Z, et al. (2013) Nucleocapsid of Tomato spotted wilt tospovirus forms mobile particles that traffic on an actin/endoplasmic reticulum network driven by myosin XI-K. New Phytol 200: 1212-1224.
9. Wu X, Li L, Jiang H (2016) Doa1 targets ubiquitinated substrates for mitochondria-associated degradation. J Cell Biol 213: 49-63.
