# Supplementary material for: Recruitment of Vps34 PI3K and enrichment of PI3P phosphoinositide in the viral replication compartment is crucial for replication of a positive-strand RNA virus
Source: PLoS Pathog. 2019 Jan 9;15(1):e1007530. doi: 10.1371/journal.ppat.1007530 (PMC6342326; doi:10.1371/journal.ppat.1007530)
Supplement: S1 Table — (PDF) [file ppat.1007530.s009.pdf]

S1 Table. List of primers and constructs used in this study

Clone	Primer		Primer sequence (from 5' to 3')	Procedure
pRS315-Vps34-Flag	F	5104	GGAAGATCTAAAATGTCACTGAACAACATAACATTCTGTG	PCR-amplified Vps34 fragment was digested with BglII&PstI, inserted into pRS315-cFlag cut with BamHI&PstI.
	R	5105	GCCCTGCAGGGTCCGCCAGTATTGTGCC	
pRS315-Vps34[N736K]-Flag	F	5104	GGAAGATCTAAAATGTCACTGAACAACATAACATTCTGTG	Vps34[N736K] fragment was PCR-amplified by overlap PCR and digested with BglII&PstI, inserted into pRS315-cFlag cut with BamHI&PstI.
	R	5105	GCCCTGCAGGGTCCGCCAGTATTGTGCC	
	F	6885	CGGCGATAGGCATTTAGACAAGTTACTAGTCACGCCAGATGGGC	
	R	6886	TTGTCTAAATGCCTATCGCCG	
pRS315-Vps34[D749E]-Flag	F	5104	GGAAGATCTAAAATGTCACTGAACAACATAACATTCTGTG	Vps34[D749E] fragment was PCR-amplified by overlap PCR and digested with BglII&PstI, insert into pRS315-cFlag cut with BamHI&PstI.
	R	5105	GCCCTGCAGGGTCCGCCAGTATTGTGCC	
	F	6887	GGGCACTTTTTCACGCAGAGTTTGGTTATATCTTGGGTCAGGACC	
	R	6888	TCTGCGTGAAAAAGTGCCC	
pTRV2-NbVps34	F	7182	CCGCTCGAGATTCAGCGAGTTGATTGGCT	PCR-amplified NbVps34 fragment was digested with BamHI&XhoI, and inserted into pTRV2 cut with BamHI&PstI.
	R	7183	CGGGATCCAATTGATTCCCACTTGTGCAT	
pRS315-Vps34-RFP	F	5104	GGAAGATCTAAAATGTCACTGAACAACATAACATTCTGTG	PCR-amplified Vps34 fragment was digested with BglII&PstI, and inserted into pRS315-cRFP cut with BamHI&PstI.
	R	5105	GCCCTGCAGGGTCCGCCAGTATTGTGCC	
pGD-AtVps34-GFP	F	5667	CGCGGATCCATGGGTGCGAACGAGTTTCGTTTC	PCR-amplified AtVps34 fragment was digested with BamHI&SmaI, and inserted into pGD-cGFP cut with BamHI&SmaI.
	R	5668	ACGCCAGTATTGAGCCCATCTGTG	
pGD-nYFP-AtVps34	F	5667	CGCGGATCCATGGGTGCGAACGAGTTTCGTTTC	PCR-amplified AtVps34 fragment was digested with BamHI&SalI, and inserted into pGD-nYFP cut with BamHI&SalI.
	R	6984	CCGACGTCGACTCAACGCCAGTATTGAGCCCATCT	
pGD-AtVps34-Flag	F	5667	CGCGGATCCATGGGTGCGAACGAGTTTCGTTTC	PCR-amplified AtVps34 fragment was digested with BamHI&SmaI, and inserted into pGD-cFlag cut with BamHI&SmaI.
	R	5668	ACGCCAGTATTGAGCCCATCTGTG	
pEsc-Vps34-3xHA	F	6922	AAGGAAAAAAGCGGCCGCATGTCACTGAACAACATAACATTCTGT	3xHA tag was digested with NotI&PacI, and inserted into pEsc-URA-NotI&PacI; PCR-amplified Vps34 was digested with NotI&BglII, and inserted into pEsc-3xHA-NotI&BglII.
	R	7018	GAAGATCTGGTCCGCCAGTATTGTGC C	
	F	7016	GAAGATCTATGGGTTACCCATACGATGTTC	
	R	7017	CCTTAATTAATCACTACTGCAGAGCAGCGTAATCTGGAACGT	
pRS425-Cup1-Flag-Ymr1	F	6903	AACTGCAGATGGAGTACATCAAGATTGCCAAA	PCR-amplified Ymr1 fragment was digested with PstI&XhoI, and inserted into pRS425-Cup1-nFlag cut with PstI&XhoI.
	R	6904	CCGCTCGAGTCACTTTCTATTAAACATATCAAATCCAAAA	
pGD-Flag-AtMtm1	F	7196	CGGGATCCATGACGCCGCCGAGA CCAC	PCR-amplified AtMtm1 fragment

	R	7316	CGACGTCGACTCATTTAGGTTGGAAATAGCTATCGTAGATG	was digested with BamHI&SalI, and inserted into pGD-nFlag cut with BamHI&SalI.
pRS425-Cup1-RFP-Flag-2xFYVE	F	6033	CCGTGATCAGAAAGTGATGCCATGTTCGCTGC	PCR-amplified 2xFYVE fragment was digested with BclI&XhoI, and inserted into pRS425-Cup1-RFP-Flag-BamHI&SalI.
	R	6034	GCGCTCGAGTCATGCCTTCTTGTTCAGCTGCTC	
	F	6115	CGGGGTACCGAAAGTGATGCCATGTTCGC	
	R	6116	CGGGGTACCTTGTCCTTGTGCCTTCTTGTTCAGCTGC	
pRS425-Cup1-RFP-Flag-PX	F	6029	CGCGGATCCGCAGCTAATTCTGTAGGGAAAATG	PCR-amplified PX fragment was digested with BamHI&XhoI, and inserted into pRS425-Cup1-RFP-Flag-BamHI&SalI.
	R	6030	GCGCTCGAGTTACTTTGACAACTGCAGGAAGTC	
pRS425-Cup1-RFP-Flag-PXm	F	6029	CGCGGATCCGCAGCTAATTCTGTAGGGAAAATG	PCR-amplified PXm fragment was digested with BamHI&XhoI, and inserted t into pRS425-Cup1-RFP-Flag-BamHI&SalI.
	R	6030	GCGCTCGAGTTACTTTGACAACTGCAGGAAGTC	
pRS425-Cup1-2xFlag-Vps34	F	7028	GAAGATCTGATTACAAGGATGACGACGATAAGATGTCACTGAACAACATAACATTCTG	PCR-amplified Vps34 fragment was digested with BglII&PstI, and inserted into pRS425-Cup1-nFlag cut with BamHI&PstI.
	R	7029	AAACTGCAGTCAGGTCCGCCAGTA TTGTG	
pRS425-Cup1-2xFlag-Vps34[N736K]	F	7028	GAAGATCTGATTACAAGGATGACGACGATAAGATGTCACTGAACAACATAACATTCTG	PCR-amplified Vps34[N736K] fragment was digested with BglII&PstI, and inserted into pRS425-Cup1-nFlag cut with BamHI&PstI.
	R	7029	AAACTGCAGTCAGGTCCGCCAGTA TTGTG	
pRS425-Cup1-2xFlag-Vps34[D749E]	F	7028	GAAGATCTGATTACAAGGATGACGACGATAAGATGTCACTGAACAACATAACATTCTG	PCR-amplified Vps34[D749E] fragment was digested with BglII&PstI, and inserted into pRS425-Cup1-nFlag cut with BamHI&PstI.
	R	7029	AAACTGCAGTCAGGTCCGCCAGTA TTGTG	
pGEX-Vps34	F	5104	GGAAGATCTAAAATGTCACTGAACAACATAACATTCTGTG	Vps34 fragment was digested with BglII&SalI, and inserted into pGEX cut with BamHI&XhoI.
	R	7668	GCCGACgtcgacTCAGGTCCGCCAGTATTGTG	
pGEX-Vps34[D749E]	F	5104	GGAAGATCTAAAATGTCACTGAACAACATAACATTCTGTG	Vps34[D749E] fragment was digested with BglII&SalI, and inserted into into pGEX cut with BamHI&XhoI.
	R	7668	GCCGACgtcgacTCAGGTCCGCCAGTATTGTG	
pYes-His-Vps21	F	5781	CgcggatccATGAACACATCAGTCACTTCCATAAAGTTG	Vps21 fragment was digested with BamHI&XhoI, and inserted into pYes2/NT cut with BamHI&XhoI.
	R	5782	ccgctcgagCTAACAACTGCAAGCACTGTTTGC	
Semi qRT-PCR	F	6997	GACATCTTCTGTCTGGCCTCC	RT-PCR was used to test NbVps34 mRNA level upon TBSV infection.<br>Check NbVps34 mRNA level in NbVps34-silenced plant.
	R	6998	CAGTTGCCAATACTCTGTATGGAG	
TBSV probe	F	1166	ATTCCTGTTTACGAAAGTTAGGT	
	R	22	GTAATACGACTCACTATAGGGCTGCATTTCTGCAATGTTCC	
CIRV probe	F	979	GGACGGAAGCTTCACTGCACAGAGT	
	R	978	TAATACGACTCACTATAGGGCTGCATTTCTGCAATGTTC	
CLSV probe	F	977	GTGGGAATGGTACCTTAATTGGTGA	

	R	976	TAATACGACTCACTATAGGGCTAGCGTGTTTCCACGCA
TCV probe	F	975	GATCCAAAGCTTGTCGATTTCGGCA
	R	974	TAATACGACTCACTATAGGGCAGGCCCCCCCCCCGCGCGA
RCNMV RNA1 probe	F	3043	AGGGGAACACGCAGTCTC
	R	3044	TAATACGACTCACTATAGGATTTTGTTTTACCAGAGGTATGC
NoV RNA1 probe	F	3867	CGGGTCCGCCAGCTAAAACAACAG
	R	3868	GTAATACGACTCACTATAGACCACTGGCATAAGCCTAGTTCG
pGAD-Cup1-His-p92	[1]		
pGAD-Cup1-Flag-p92	[1]		
pGBK-Cup1-His-p33-Gal1-DI72	[1]		
pGBK-Cup1-Flag-p33-Gal1-DI72	PROVIDED BY DR. J. POGANY (U. KENTUCKY)		
pYes-His-p92	[2]		
pEsc-His-p33/DI72	[2]		
pYes-His-CNV p92	[3]		
pEsc-His-CNV p33-DI72	[3]		
pEsc-RFP-p33/DI72	[4]		
pEsc-GFP-p33.DI72	[4]		
pYes-strep-p95	[4]		
pEsc-strep-p36/DI72	[4]		
pEsc-GFP-p36/DI72	[4]		
pEsc-His/Cupm/NoV/RNA1/TRSVrz	[5]		
pRS315-cFLAG	[4]		
pRS315-Pex13-GFP	[4]		
pRS315-Pex13-BFP	[4]		
pRS315-GFP-2Xfyve	[4]		
pRS315-RFP-2Xfyve	[4]		
pRS425-Cup1-Flag	[4]		
pTRV2-cGFP	[4]		
pGD-RFP-SKL	[4]		
pGD-CoxIV-RFP	[6]		
pGD-p33-BFP	[4]		
pGD-p33-RFP	[4]		
pGD-p33-cYFP	[4]		
pGD-p36-cYFP	[4]		
pGD-nYFP-MBP	[4]		
pGD-GFP-AtRab5B	[4]		
pGD-RFP-2xFYVE	[4]		

References:

1. Barajas D, Li Z, Nagy PD (2009) The Nedd4-type Rsp5p ubiquitin ligase inhibits tombusvirus replication by regulating degradation of the p92 replication protein and decreasing the activity of the tombusvirus replicase. J Virol 83: 11751-11764.

2. Xu K, Huang TS, Nagy PD (2012) Authentic in vitro replication of two tombusviruses in isolated mitochondrial and endoplasmic reticulum membranes. J Virol 86: 12779-12794.
3. Xu K, Lin JY, Nagy PD (2014) The hop-like stress-induced protein 1 cochaperone is a novel cell-intrinsic restriction factor for mitochondrial tombusvirus replication. J Virol 88: 9361-9378.
4. Xu K, Nagy PD (2016) Enrichment of Phosphatidylethanolamine in Viral Replication Compartments via Co-opting the Endosomal Rab5 Small GTPase by a Positive-Strand RNA Virus. PLoS Biol 14: e2000128.
5. Lin JY, Mendu V, Pogany J, Qin J, Nagy PD (2012) The TPR Domain in the Host Cyp40-like Cyclophilin Binds to the Viral Replication Protein and Inhibits the Assembly of the Tombusviral Replicase. PLoS Pathog 8: e1002491.
6. Kohler RH, Zipfel WR, Webb WW, Hanson MR (1997) The green fluorescent protein as a marker to visualize plant mitochondria in vivo. Plant J 11: 613-621.
